# Combination treatment with acupoint therapy and conventional medication for non-motor symptoms in Parkinson’s disease: a systematic review and meta-analysis

**DOI:** 10.3389/fneur.2025.1381500

**Published:** 2025-05-22

**Authors:** Wenjiao Hu, Hao Zhou, Wenwen Zhu, Songcheng Xie, Yue Zeng, Zhengyan Wang

**Affiliations:** ^1^Chengdu University of Traditional Chinese Medicine, Chengdu, China; ^2^Sichuan Integrative Medicine Hospital, Chengdu, China

**Keywords:** Parkinson’s disease, acupoint therapy, non-motor symptom, randomized controlled trials, meta-analysis

## Abstract

**Background:**

Managing Parkinson’s non-motor symptoms (PD-NMS) is challenging. Traditional methods have limited efficacy for NMS. Acupoint therapy offers a safe and personalized option. There has been a growing number of studies on acupoint therapies for PD-NMS. However, a systematic review of their effectiveness and safety is currently not available. Therefore, this study aims to evaluate the effectiveness and safety of acupoint therapy for PD-NMS.

**Methods:**

PubMed, Web of Science, Cochrane Library, Embase, China National Knowledge Infrastructure, Wanfang Database, China Science and Technology Journal Database, and Chinese Biomedical Literature Database were searched. Data were analyzed using fixed or random effects models. Randomized controlled trials (RCT) in Chinese and English relating to acupoint therapy for NMS in PD (PD-NMS), individuals diagnosed with PD, primary and secondary outcome measures are HAMD, MMSE, MoCA, PDSS, PSQI were included. Cochrane risk-of-bias tool (ROB 2) for RCT was used. Meta-analyses were performed to calculate the RR and WMD. Subgroup analyses, sensitivity analyses, and funnel plot analyses were conducted.

**Results:**

Forty-two studies (3120 PD-NMS patients) from database establishment to May 2023 were included. According to the ROB 2 evaluation, risks of bias in random sequence generation, missing data and selective reporting were low, moderate in allocation concealment, and high in blinding. Acupoint therapy combined with CM or NDT was more effective than single-therapy in most outcome measures like effective rate, HAMD, MoCA, PDSS and PSQI, but the MMSE difference was non-significant.

**Conclusion:**

Due to the large number of research subjects, differences in the severity of the diseases, and possible variations of the intervention details, there is a certain degree of heterogeneity in the research results. However, acupoint therapy or acupoint therapy combined with CM could be an option for the treatment of PD-NMS in the future.

**Systematic review registration:**

This review protocol has been registered in PROSPERO (ID: CRD42023426305). https://www.crd.york.ac.uk/PROSPERO/.

## Introduction

Parkinson’s disease (PD) is currently conceptualized as a complex neuropsychiatric disorder resulting from a reduction in the number of dopamine-producing cells in the substantia nigra (1). This disease is a common condition; in 2016, approximately 6.1 million people worldwide were affected (2, 3). Although the cause of PD remains unknown, various genetic factors have been identified (4). PD is currently described as a multisystem neurodegenerative disease because it simultaneously involves the central, enteric, and autonomic nervous systems; adaptive immune system; and gastrointestinal tract (5). Clinically, PD is characterized by the occurrence of both motor symptoms and non-motor symptoms (PD-NMS) (6–10). PD-related NMS (PD-NMS) represent a major source of disability, which may exceed the burden from PD-related motor impairments (11, 12). Furthermore, PD-NMS such as hyposmia/anosmia, rapid eye movement sleep behavior disorder, depression, anxiety, cardiac sympathetic denervation, and constipation may precede dopaminergic neuronal loss—and the consequent motor symptoms—by several years (13, 14).

Currently, the first-line therapy for patients with PD is either daily oral levodopa or a dopamine agonist. Levodopa offers good control of motor symptoms but does not modify disease evolution (11, 15). The effectiveness of pharmacological treatments tends to wear off over time, and patients need to take increasingly higher drug doses, thus imposing a heavy burden on families and society (16). Levodopa may induce or exacerbate the psychiatric symptoms in PD patients: such as hallucinations, delusions, depression, anxiety. Dopamine agonists have no significant effect on improving cognitive and sleep disorders and are associated with more side effects (e.g., nausea, orthostatic symptoms, and sleep disturbances) (17). In particular, neuropsychiatric PD-NMS may severely alter the quality of life of both patients and their caregivers (18, 19). Given the limitations of pharmacological therapy, either in combination with or as an alternative to dopamine replacement therapy, complementary and alternative medicine and integrative medicine approaches are widely used to improve brain and overall health in people with PD (20–22).

For example, lifestyle modifications can provide therapeutic benefits for patients with PD. Aerobic exercise continues to be a key treatment that is potentially neuroprotective in PD (23–25). Exercise promotes substances that have been termed “exerkines” which can influence homeostasis (26, 27). A clinical study confirmed long-term Tai Chi training can improve motor function, especially gait and balance in PD (28). Other complementary alternative therapies, such as thermal rehabilitative treatments statistically improved motor function, balance, QoL, and psychological well-being (29). Traditional Chinese medicine (TCM) has also been widely applied to improve PD symptoms and control disease evolution (18, 30, 31).

Acupoint therapy is an essential part of TCM and has many validated treatment effects. It includes many kinds of alternative treatments, such as acupuncture, moxibustion, massage, acupoint injection, acupressure, and cupping. These therapies can be considered complementary and alternative medicine (22, 32, 33). These treatments can have direct and positive effects on the body, both on motor symptoms and PD-NMS, when an acupoint is stimulated (34). Some studies have reported that acupuncture stimulation can inhibit an increase in *α*-synuclein and boost the survival rate of dopaminergic neurons in the substantia nigra (35, 36). Moreover, acupuncture can improve gut microbial dysregulation and inhibit neuroinflammation in the substantia nigra and striatum, all of which are associated with a PD phenotype (35). Acupuncture also inhibits neuroinflammation and gut microbial dysbiosis in a mouse model of PD. Similarly, the stimulation of acupoints such as CV-12, RN-7, ST-36, and LR-3 via electroacupuncture reportedly regulates brain/gut levels of peptides such as neuropeptide Y, cholecystokinin, gastrin, and peptide YY, thereby reducing neuronal apoptosis, the nigral inflammatory response, and oxidative stress in the substantia nigra of PD model rats (37).

Notably, most of the published reviews on acupoint therapy for PD have focused on motor symptoms rather than PD-NMS. In addition, few reviews have systematically summarized the efficacy of different types of acupuncture in various functional disorders in PD. Researchers have also often overlooked the different categories of acupuncture and other types of acupoint therapy, thus neglecting the importance of acupuncture points in treating disease. We have therefore conducted a systematic review and meta-analysis of the available literature to determine the effects of acupoint therapy combined with conventional medication (CM) on the regulation of PD-NMS. Our study included different types of acupoint interventions for these patients. And CM therapies include Chinese medicine and western medicine.

## Methods and analysis

### Protocol and registration

The study was conducted according to the Preferred Reporting Items for Systematic Reviews and Meta-Analyses (PRISMA) for systematic review protocols (38) and meta-analysis checklist (39). This review protocol has been registered in PROSPERO (ID: CRD42023426305).

### Search methods

An electronic literature search was performed using the following databases for articles published in English and Chinese as of May 2023: PubMed, Web of Science, Cochrane Library, Embase, China National Knowledge Infrastructure, Wanfang Data, China Science and Technology Journal Database, and Chinese Biomedical Database. Medical Search Heading (MeSH) terms and their synonyms (free text) were combined as search terms using the Boolean operators “AND” and “OR.” The following MeSH terms were used: “Parkinson Disease,” “Acupuncture,” “Acupressure,” “Massage,” “Cupping Therapy,” “moxibustion,” “acupoint,” “acupoint injection,” “auricular acupuncture,” “scraping therapy,” and “randomized controlled trial.” The detailed search strategy is shown in the box. For more details in Supplementary material 3.

Search items in PubMed.

**Table 1:** 

No.	Search terms
#1	Acupoint. Mesh
#2	Acupuncture Points. Mesh
#3	(Acupuncture Point) or (Point, Acupuncture) or (Acupoints) or (Acupoint). ti. ab
#4	Acupuncture. Mesh
#5	Acupuncture. ti. ab
#6	Acupressure. Mesh
#7	Acupressure. ti. ab
#8	Acupuncture, auricular. Mesh
#9	(Acupunctures, Ear) or (Ear Acupunctures) or (Auricular Acupuncture) or (Acupuncture, Auricular) or (Auricular Acupunctures). ti. ab
#10	Massage. Mesh
#11	Massage. ti. ab
#12	(Manipulations, Musculoskeletal) or (Manipulation Therapy) or (Manipulative Therapies) or (Therapies, Manipulative) or (Therapy, Manipulation) or (Manipulation Therapies) or (Manual Therapies) or (Therapy, Manual). ti. ab
#13	Cupping therapy. Mesh
#14	(Cupping Therapies) or (Therapy, Cupping) or (Cupping Treatment) or (Treatment, Cupping). ti. ab
#15	Moxibustion. Mesh
#16	Moxibustion. ti. ab
#17	Acupoint injection. Mesh
#18	Acupoint injection.ti. ab
#19	#1 or #2–18
#20	Randomized controlled trial. Mesh
#21	Controlled clinical trial. ti. ab
#22	Randomized. ti. ab
#23	Randomly. ti. ab
#24	Trial. ti. ab
#25	#20 or #21–24
#26	Parkinson’s disease. Mesh
#27	Parkinson’s disease. ti. ab
#28	Parkinsonian disorders. ti. ab
#29	#26 or #27–28
#30	#19 and #25 and #29

### Criteria for consideration of studies in this review

#### Types of studies

RCTs on the combination treatment of acupoint therapy and CM for PD were included in the present review. Protocols, case reports, reviews, letters, cell experiments, animal experiments, and non-RCTs were excluded.

#### Types of participants

Individuals diagnosed with PD, with no restrictions on age, sex, race, or disease duration.

#### Types of interventions

Experimental groups with all types of acupoint therapy (including acupuncture, moxibustion, acupressure, massage, acupoint injection, cupping, skin scraping, transcutaneous acupoint electrical stimulation, and acupotomy) were included. The different types of acupoint therapy were then analyzed again as different interventions.

#### Types of comparisons

Control groups that received CM, which was defined as all types of anti-PD drugs used alone or in combination, were included. Control groups were also able to receive CM combined with a sham intervention.

To summarize, we included and classified the following comparisons:

1. Acupoint therapy combined with CM versus CM used alone or in combination.
2. Acupoint therapy combined with CM versus CM combined with a sham intervention.

#### Types of outcome measures

We extracted effect sizes at the first time point after the end of interventions. In the current review, we only included RCTs that reported the following main outcomes (i.e., that assessed PD-NMS using effective and validated scales): total effective rate, Hamilton Depression Scale (HAMD), Mini-Mental State Examination Scale (MMSE), Montreal Cognitive Assessment Scale (MoCA), PD Sleep Scale (PDSS), Pittsburgh Sleep Quality Index (PSQI), and Self-Assessment of Quality of Life for Constipation Patients (PAC-QoL). Only Chinese and English articles that met the aforementioned inclusion criteria were included.

### Study selection process

Two independent authors (WH and HZ) revised the titles and abstracts of the searched papers to determine suitable studies. Then, the two authors revised the full texts of the retrieved reports independently. Any conflicts between authors were solved by the third author (ZW).

### Data extraction

Two independent authors (WH and HZ) extracted data. The following data were extracted in a predefined data collection form: first author, year of publication, language, sample size, demographic data of participants, baseline characteristics of patients, diagnostic criteria, inclusion and exclusion criteria for participants, experimental and control interventions, course of treatment, frequency, location of the study, outcomes, and safety. Any conflicts between authors were solved by the third author (ZW).

### Quality assessment

The Cochrane risk-of-bias tool (ROB 2) for RCTs was used to assess potential bias in the included studies (40). This risk-of-bias tool consists of the following seven domains: (I) random sequence generation, (II) allocation concealment, (III) blinding of participants and personnel, (IV) blinding of outcome assessment, (V) incomplete outcome data, (VI) selective reporting, and (VII) other bias (e.g., we assessed trials with no reported monitoring of self-acupressure procedures as having a high risk of compliance bias). Each RCT was categorized as having a low, high, or unclear risk of bias in each domain.

### Data synthesis and statistical analysis

Review Manager (RevMan, version 5.4; The Nordic Cochrane Centre, The Cochrane Collaboration, Copenhagen, Denmark) and STATA (version 17.0) software were used to quantitatively analyze the included studies, as follows. (I) Combination of effects: continuous outcomes (HAMD, MMSE, PDSS, MoCA, PSQI, and PAC-QoL) were evaluated using weighted mean differences (WMDs), whereas dichotomous outcomes (rates of improvement) were assessed using risk ratios (RRs). The 95% confidence interval (CI) was evaluated for all effect sizes, with a 95% CI excluding the point of no effect, indicating significance. When the median and first and third quartiles were provided, the mean and standard deviation were estimated using the formula developed by Wan et al. (41). (II) Heterogeneity test: the *Q*-test was performed to assess heterogeneity among studies. If *p* > 0.10, the results of multiple similar studies were considered homogeneous. If *p* > 0.10 and *I*^2^ ≥ 0 and ≤ 50%, a fixed effects model was used for the integrative analysis of studies. If *p* ≤ 0.10 or *I*^2^ > 50%, the results of multiple similar studies were considered heterogeneous, and a sensitivity analysis was performed (42). Articles were removed sequentially to observe changes in heterogeneity, WMDs, and RRs. If heterogeneity was altered after the removal of an article, it was considered a source of heterogeneity, and the underlying reason was analyzed. In contrast, if heterogeneity remained unaltered, a random effects model was used for a more conservative evaluation of the intervening effects. Subgroup analyses were performed based on acupoint methods (single acupuncture versus single moxibustion versus acupuncture combined with other therapies). When ≥10 studies were included in the meta-analysis, Egger’s test was used to assess publication bias, with *p* < 0.05 indicating significance (43).

### Ethical considerations

Although the studied specimens were human, this is a secondary analysis of published articles and does not involve ethical issues.

## Results

### Study selection

**Figure 1:**
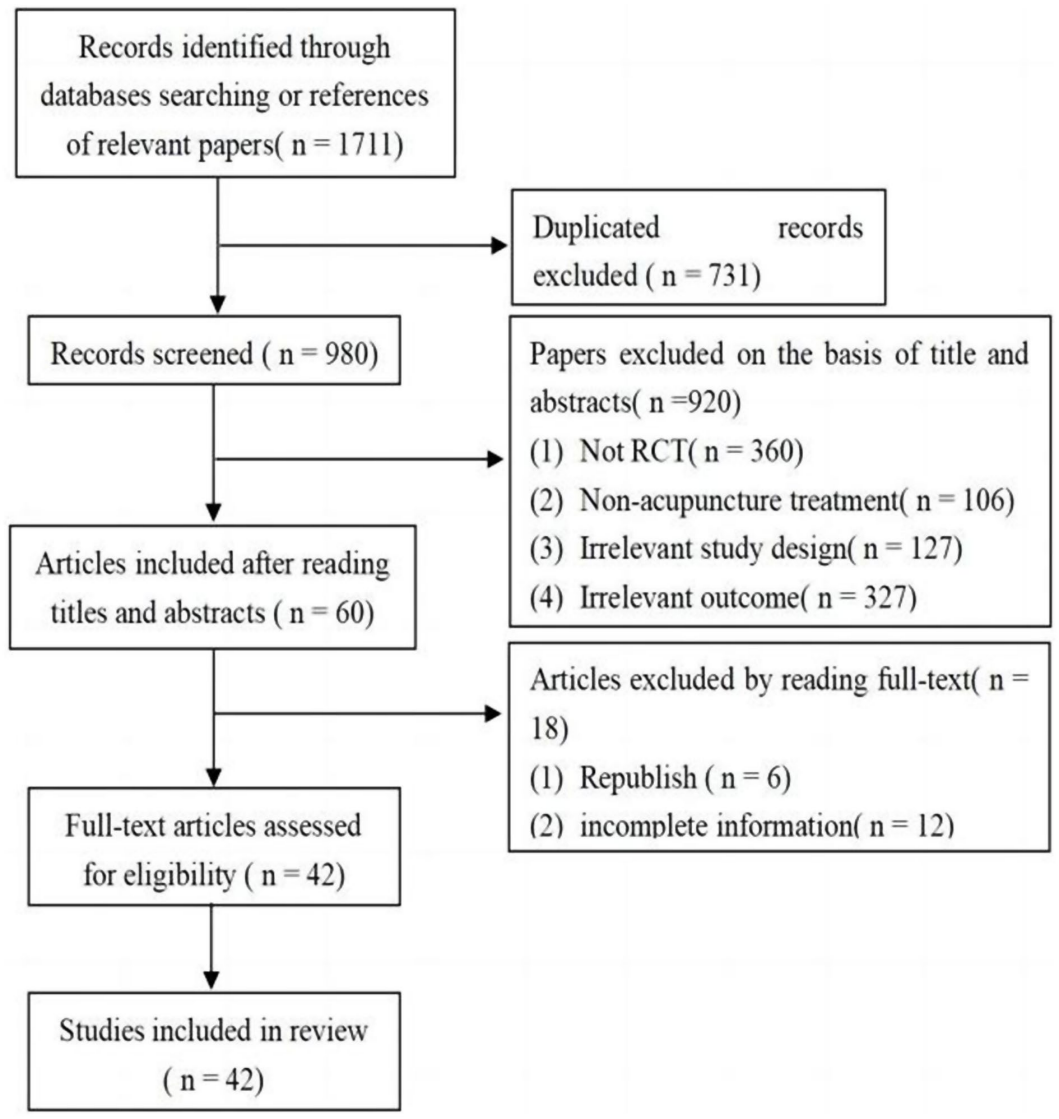
Literature screening process.

From the eight databases, 1711 articles were selected. Of these, 731 articles were removed as duplicates. Of the remaining 980 articles, many articles were excluded because they failed to meet the inclusion criteria. Finally, 60 full-text articles that met the eligibility criteria were selected. According to the inclusion and exclusion criteria, 42 articles were eventually included in the review (44–85). The study selection process is illustrated in Figure 1.

### Study characteristics and study quality

**Figure 2:**
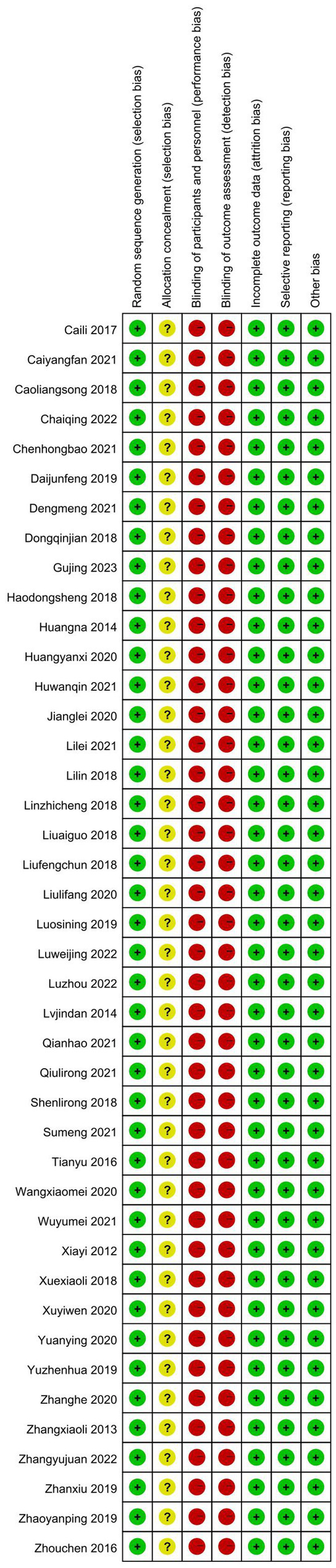
**(A)** Risk of bias summary, **(B)** Risk of bias domain for each included study.

The baseline information of the 42 studies included in this systematic review and meta-analysis is summarized in Supplementary Table 1. Of the 3,120 patients with PD, 1556 were assigned to the experimental group and 1,564 were assigned to the control group, with 1743 male patients and 1,377 female patients. Of the 42 studies, 24 were randomized into groups using the random number table method (45–49, 52–78, 81–85), two were randomized according to the order of treatment (63, 73), two were grouped by computer random sampling (69, 72), one (50) was grouped by convenient sampling selection, one was grouped by numerical random sampling (64), and one was grouped by envelope (85). The remaining 11 studies (51, 55–59, 65, 68, 77, 78, 81) did not mention the specific randomization method. Of the 42 studies, none mentioned allocation concealment. Given the specificity of acupuncture procedures, the studies displayed a high risk of bias in terms of the blinding of participants and personnel; although one study (85) used a single-blinding method, no other studies mentioned the blinding method that was used. All 42 studies reported outcomes, and no studies demonstrated attrition bias. In summary, the risk of bias in random sequence generation was low. The risk of bias of allocation concealment was moderate. The risks of bias of blinding of participants and personnel and outcome assessment were high. The risks of bias of missing outcome data and selective reporting were low. The methodological quality of studies is shown in detail in Figure 2. A summary of the basic characteristics of the included studies is shown in Supplementary Table 2. All of the studies mentioned sample size. 41 studies mentioned the proportion of female in their research, and women were more than men in only five studies. 37 of 42 studies mentioned the disease duration, and 33 studies had patients with disease of less than 10 years.

### Meta-analysis findings

#### Effective rates

**Figure 3:**
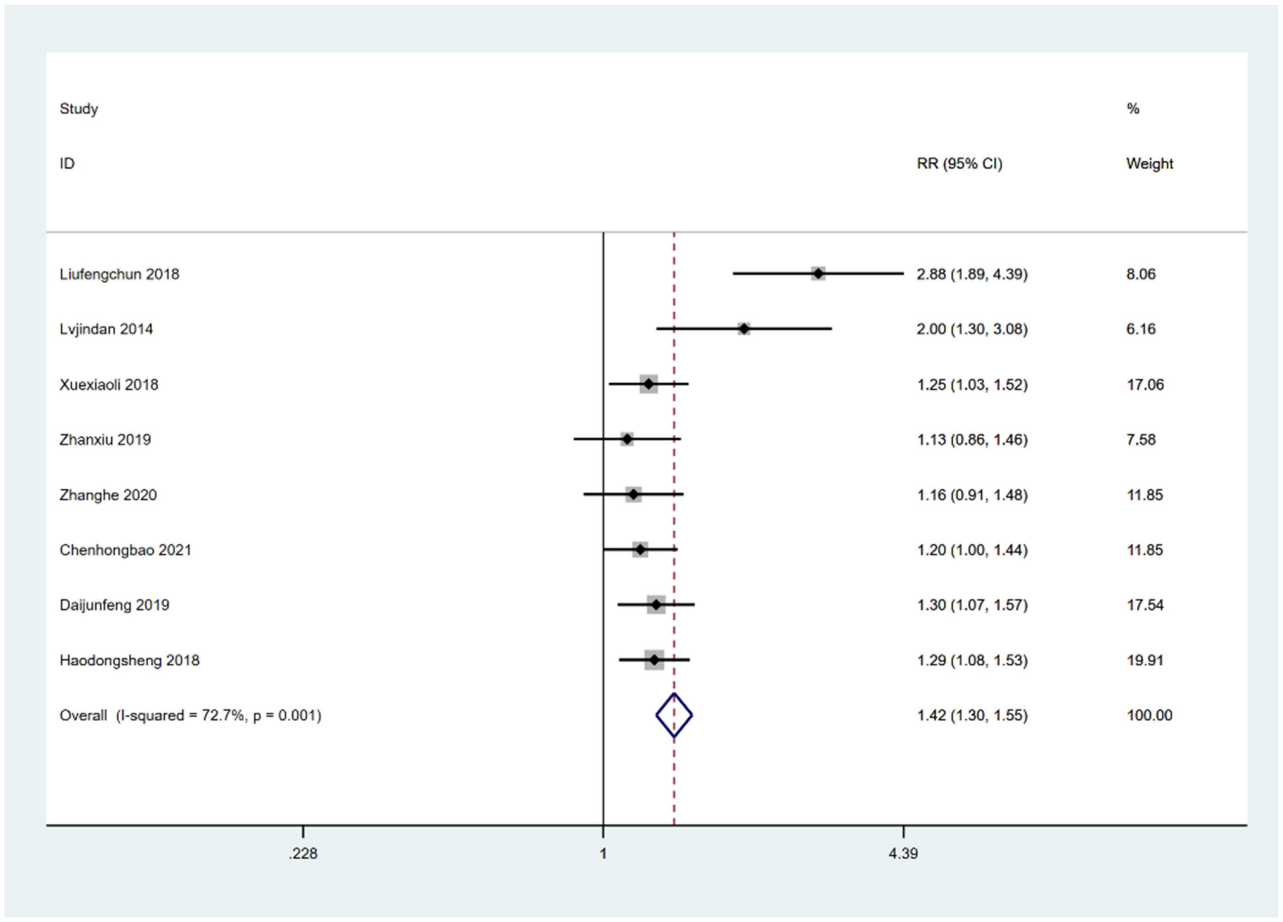
Forest plot of comparison-acupoint therapy combined with CM or non-drug therapies vs. CM or non-drug therapies alone on response rate.

Twenty-eight RCTs reported the effective rates in PD-NMS (49–58, 60, 61, 63, 65, 68, 70, 71, 73–76, 78, 81–85). The effective rates were significantly better in patients treated with acupoint therapy combined with CM or non-drug treatment (NDT) than in those treated with CM or NDT alone (RR = 1.25; 95% CI, 1.16 to 1.35; *Z* = 5.67; *p* = 0). Heterogeneity was observed among studies (chi-squared = 93.24; *df* = 27 [*p* = 0]; *I*^2^ = 71%) (Figure 3).

**Figure 4:**
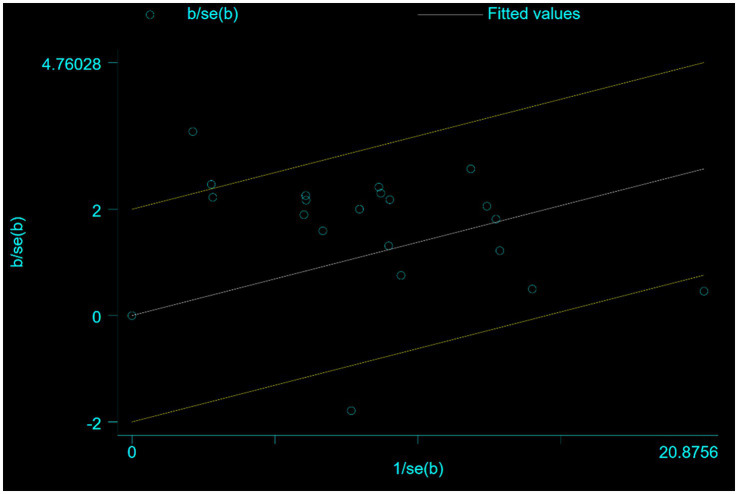
Forest plot comparing the effects of acupoint therapies plus western drugs versus western drugs alone on effective rates.

**Figure 5:**
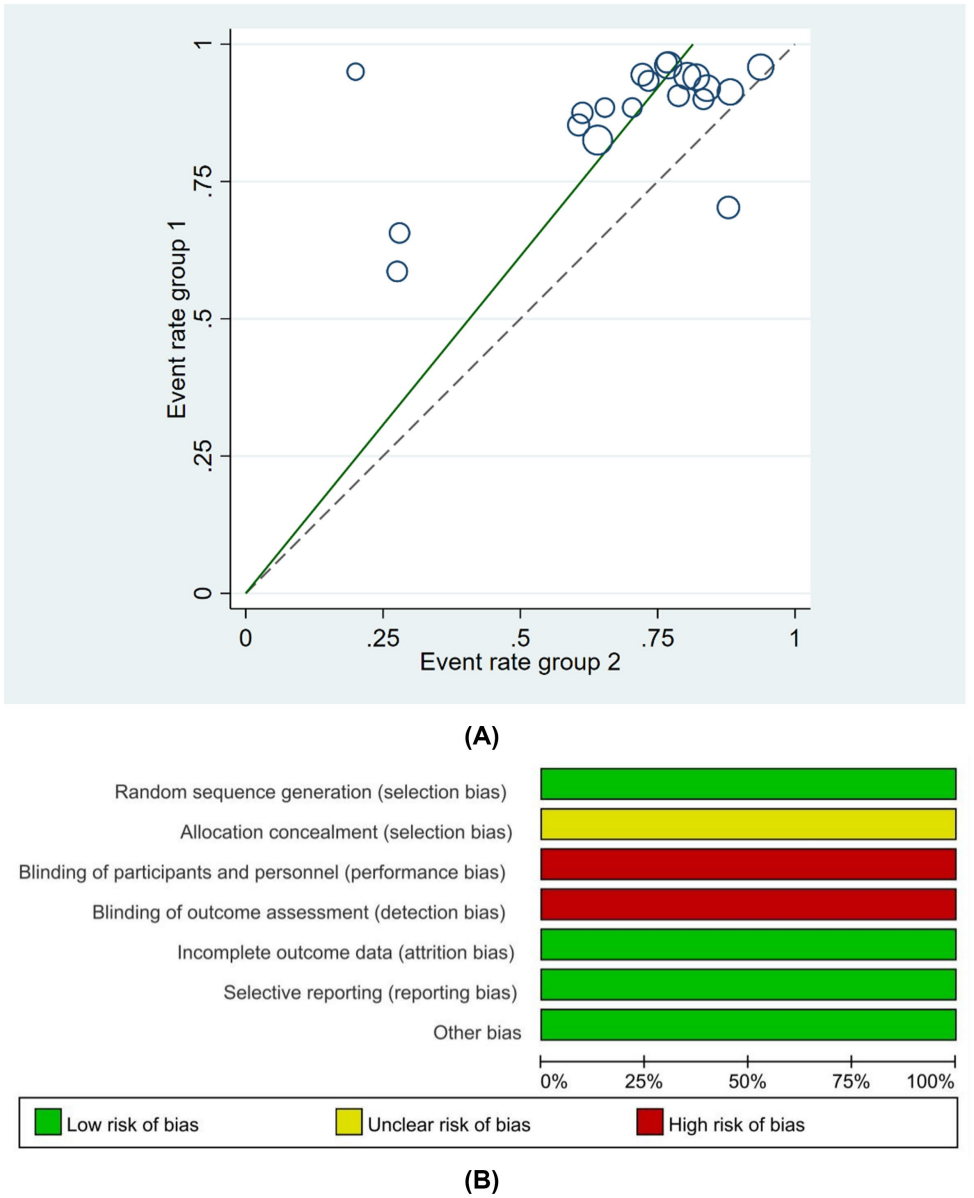
**(A)** L’Abbe plot comparing the effects of acupoint therapies plus western drugs versus western drugs alone on effective rates. **(B)** Galbraith radial plot comparing the effects of acupoint therapies plus western drugs versus western drugs alone on effective rates.

In the subgroup analysis, 20 studies (49, 52, 56, 60, 63–65, 65, 68, 70, 71, 73–76, 78, 81–85) reported a combination of acupoint therapy plus western drugs versus western drugs alone. The effective rates of a combination of acupoint therapy plus western drugs were significantly better than those of western drugs alone. A fixed effects model was used for the analysis (RR = 1.2; 95% CI, 1.11 to 1.30; *Z* = 4.38; *p* = 0; heterogeneity test: chi-squared = 56.05, *df* = 19, *p* = 0, *I*^2^ = 66%) (Figure 4). Further investigation using L’Abbe and Galbraith radial plots indicated that four articles had a strong possibility of heterogeneity (Figures 5A,B). A heterogeneity search was thus required. A sensitivity analysis of the 20 articles revealed that four studies (56, 63, 70, 84) had a relatively large influence on heterogeneity. After removing these studies, the random effects method was selected for the meta-analysis (RR = 1.15; 95% CI, 1.08 to 1.22; *Z* = 4.38; *p* < 0.00001; heterogeneity test: chi-squared = 26.64, *df* = 15, *p* = 0.032, *I*^2^ = 43.7%) (Supplementary Figure 1). A funnel plot was drawn to investigate publication bias; its symmetry indicated no publication bias (Supplementary Figure 2). However, the Egger’s bias test (with *p* < 0.05) indicated that there was publication bias in the 20 included studies (Supplementary Figure 3). The aforementioned asymmetrical funnel diagram was thus processed using the shear compensation method (Supplementary Figure 4); the seven points in the square represent the effect sizes of the literature that will need to be included in future studies. Combined with the aforementioned funnel diagram, the results of further inclusion are close to seven studies (52, 56, 63, 64, 70, 71, 84). The symmetry of the funnel plot can then be conducted, preventing publication bias.

**Figure 6:**
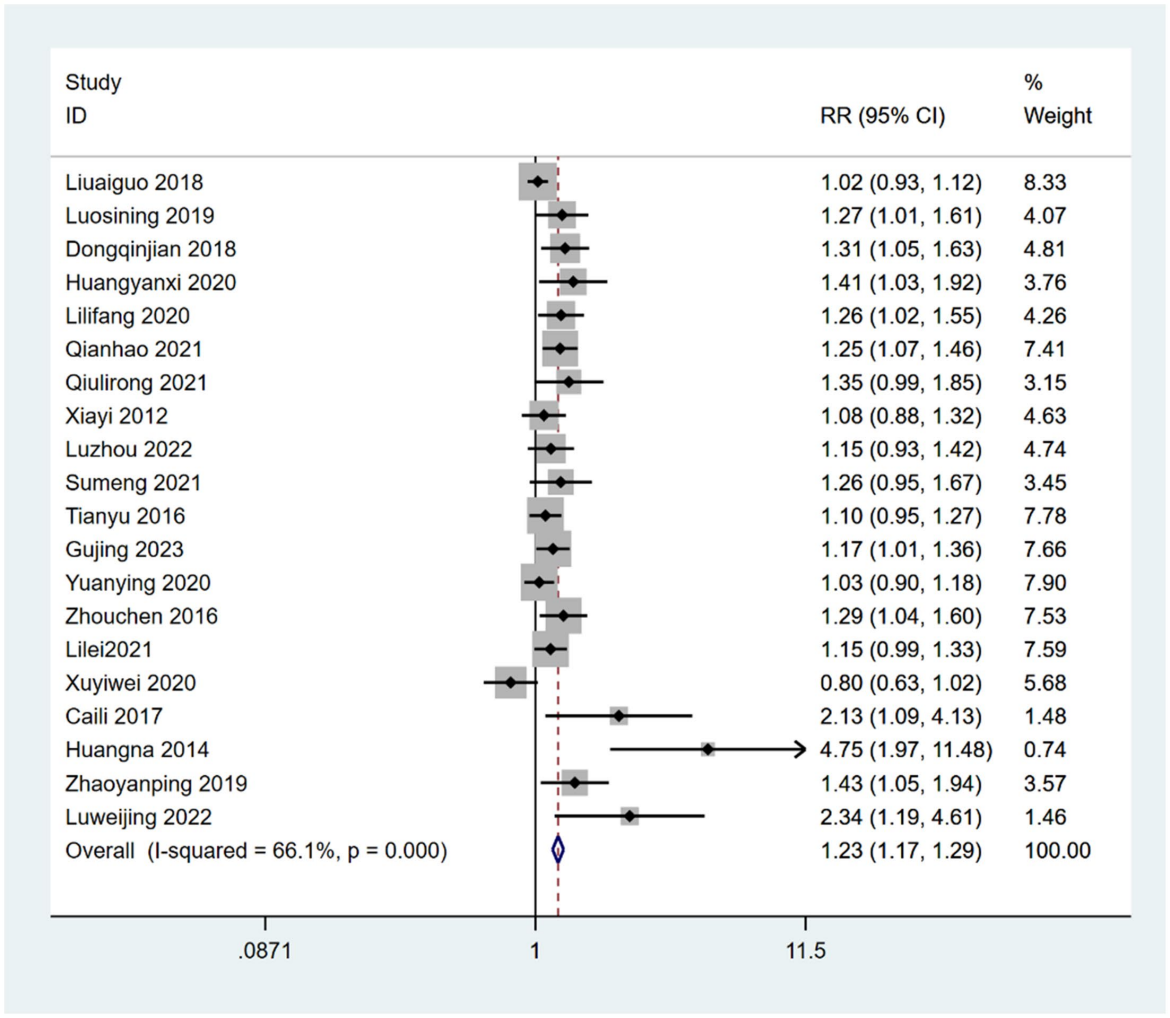
Forest plot comparing the effects of acupoint therapies plus other therapies versus other therapies alone on effective rates.

Eight studies (50, 51, 53–55, 57, 58, 61) reported comparisons between a combination of acupoint therapy plus other therapies versus other therapies alone. The effective rates of acupoint therapy combined with other therapies were better than those of other therapies alone. A fixed effects model was used for the analysis (RR = 1.42; 95% CI, 1.30 to 1.55; *Z* = 7.72; *p* = 0.0002; heterogeneity test: chi-squared = 25.67, *df* = 7, *p* = 0.0006, *I*^2^ = 72.7%) (Figure 6). Further examination of L’Abbe and Galbraith radial plots indicated a strong possibility of heterogeneity in one article (48) (Supplementary Figures 5, 6). A heterogeneity search was therefore required. A sensitivity analysis of the eight articles revealed that one article had a large impact on heterogeneity. After removing this study, the combined effect variables of the meta-analysis were large. Furthermore, a subsequent heterogeneity test revealed no heterogeneity in the remaining seven articles. A meta-analysis with fixed effects was performed after exclusion (RR = 1.29; 95% CI, 1.18 to 1.40; *Z* = 5.88; *p* < 0.001; heterogeneity test: chi-squared = 6.37, *df* = 6, *p* = 0.38, *I*^2^ = 5.8% [<50%]) (Supplementary Figure 7). This finding suggests that the efficacy of acupoint therapy combined with other therapies is significantly better than that of other therapies alone. A funnel plot was drawn to investigate publication bias; its symmetry indicated no publication bias (Supplementary Figure 8). The Egger’s bias test (*p* > 0.05) also indicated no publication bias (Supplementary Figure 9).

### HAMD scores

**Figure 7:**
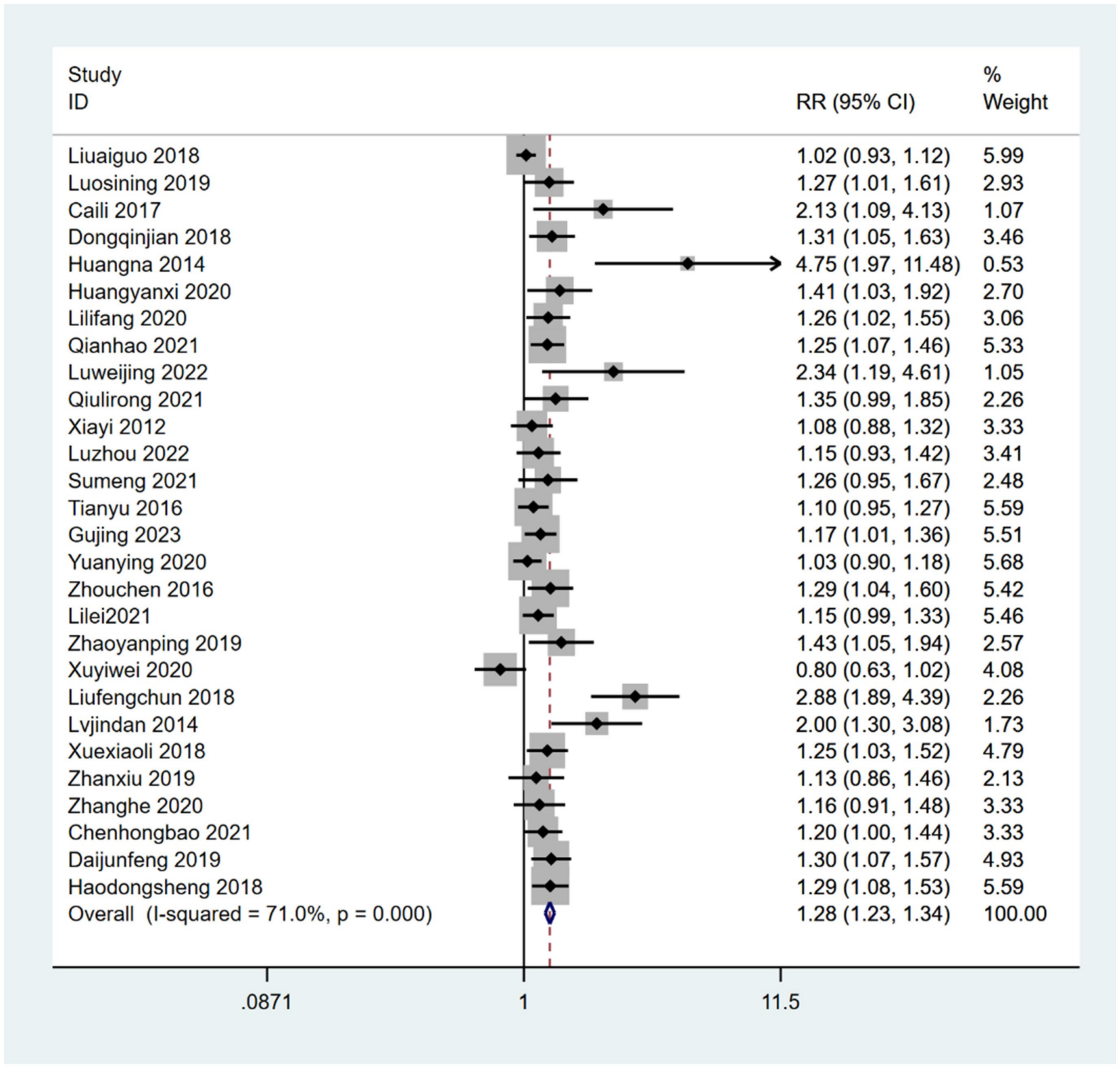
Forest plot comparing the effects of acupoint therapies plus CM or NDT versus CM or NDT alone on HAMD.

Thirteen RCTs reported the use of HAMD for assessing depression status (58, 59, 61, 65, 66, 73, 74, 76, 78–81, 84). HAMD scores were significantly better in patients treated with acupoint therapy combined with CM or NDT than in those treated with CM or NDT alone. A random effects model was used for the analysis (WMD = −2.14; 95% CI, −2.9 to −1.38; *Z* = 5.54; *p* < 0.00001). Heterogeneity was observed among the studies (chi-squared = 74.59; *df* = 12 [*p* < 0.00001]; *I*^2^ = 83.9%) (Figure 7). The results of a sensitivity analysis were not significantly different from the aforementioned results.

In the subgroup analysis, 10 RCTs (59, 65, 66, 73, 74, 76, 78, 79, 81, 84) reported comparisons between a combination of acupoint therapy with drugs versus drugs alone, and 3 RCTs (58, 61, 80) reported comparisons between acupoint therapy combined with other therapies versus other therapies alone. The fixed effects model results suggested strong heterogeneity in the 10 studies. After deleting two studies, heterogeneity still existed; a random effects model was therefore used for the analysis (WMD = −2.1; 95% CI, −3.15 to −1.05; *Z* = 3.91; *p* < 0.00001; heterogeneity test: chi-squared = 73.68, *df* = 9 [*p* = 0], *I*^2^ = 87.8%) (Supplementary Figure 10). The HAMD scores of patients treated with a combination of acupoint therapy and drugs were better than those of patients treated with drugs alone.

Acupoint therapy combined with other therapies was more effective than other therapies alone in improving HAMD scores (WMD = −2.26; 95% CI, −2.77 to −1.75; *Z* = 8.71; *p* < 0.00001; heterogeneity test: chi-squared = 0.9, *df* = 2 [*p* > 0.05], *I*^2^ = 0%) (Supplementary Figure 11). Heterogeneity was not assessed because only three studies reported comparisons between acupoint therapy combined with other therapies versus other therapies alone. Additionally, a meta-regression analysis of different acupoint therapies using “type of therapy” as an ordinal variable revealed no differences between acupuncture and moxibustion or acupoint sticking (Supplementary Figure 12).

A funnel plot was drawn to investigate publication bias; its symmetry indicated no publication bias (Supplementary Figure 13). Similarly, Egger’s test (*p* > 0.05) indicated no publication bias (Supplementary Figure 14).

### MMSE scores

**Figure 8:**
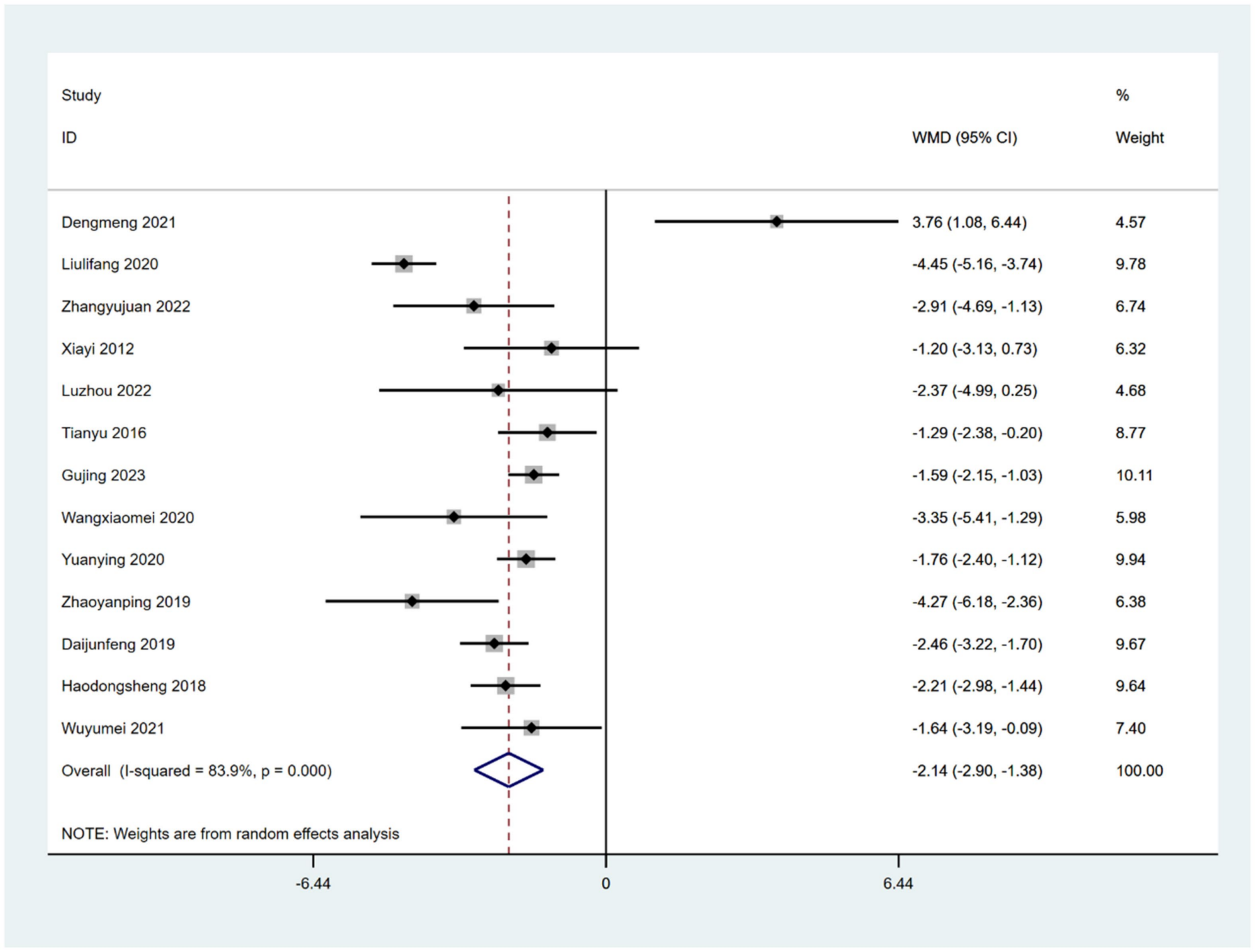
Forest plot comparing the effects of acupoint therapies with other therapies versus other therapies alone on MMSE.

Two RCTs (45, 46) reported the use of MMSE, which assesses intellectual status and cognitive function. MMSE scores appeared to be better in patients treated with a combination of acupoint therapy and other therapies than in those treated with other therapies alone, but this effect was not significant. A random effects model was used for the analysis (WMD = 2.33; 95% CI, −0.99 to 5.65; *Z* = 1.37; *p* > 0.05). Heterogeneity was observed among studies (chi-squared = 14.45; *df* = 1 [*p* = 0.0001]; *I*^2^ = 93%) (Figure 8). The results of the sensitivity analysis were not significantly different from the aforementioned results.

### MoCA scores

**Figure 9:**
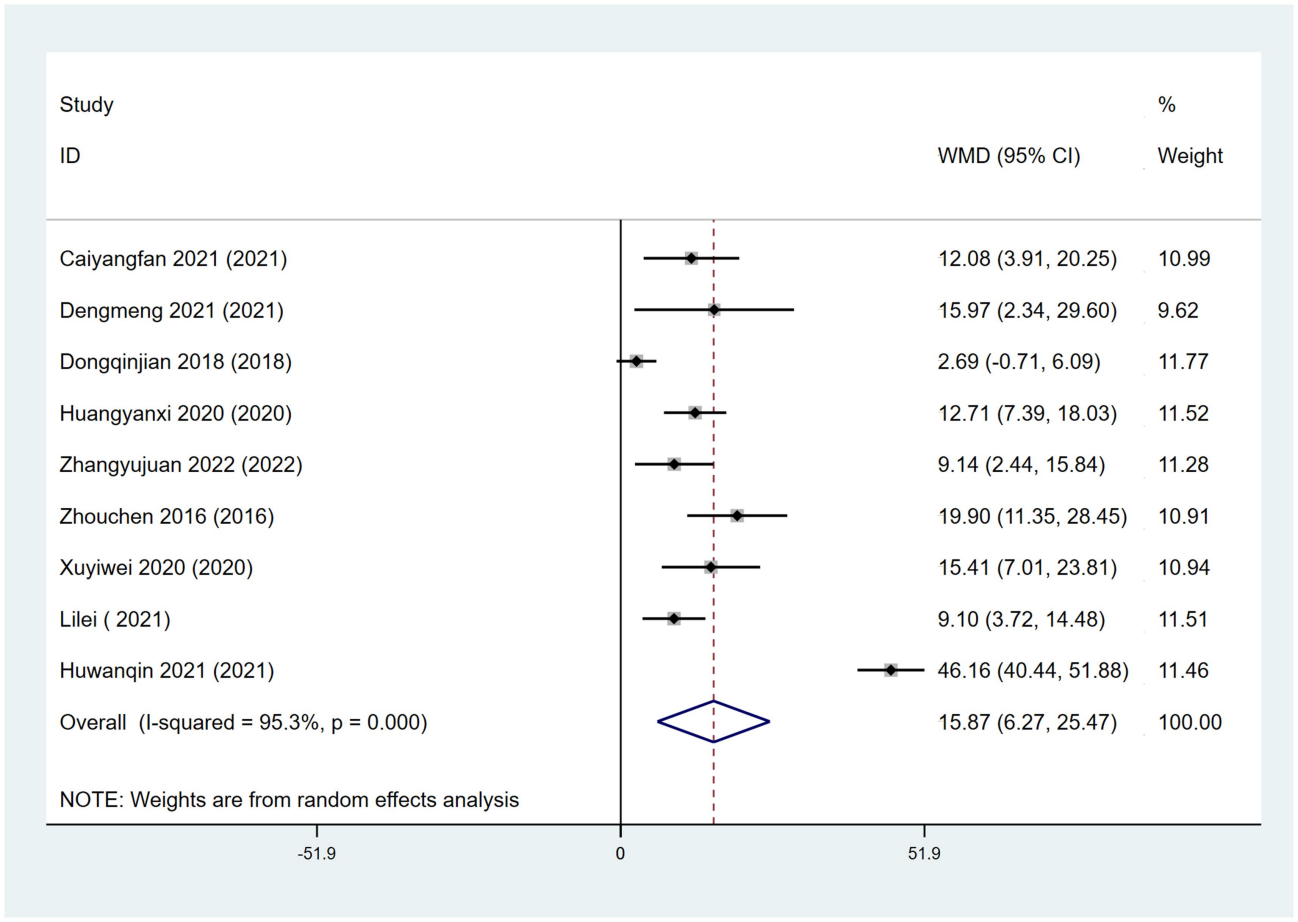
Forest plot comparing the effects of acupoint therapies with other therapies versus other therapies alone on MoCA.

Eight RCTs (45, 59, 68, 69, 71, 75, 77, 80) reported the use of MoCA, which assesses cognitive function. MoCA scores were significantly better in patients treated with a combination of acupoint therapy with other therapies than in those treated with other therapies alone. A random effects model was used for the analysis (WMD = 2.73; 95% CI, 0.63 to 4.84; *Z* = 2.54; *p* < 0.00001). Heterogeneity was observed among studies (chi-squared = 179.26; *df* = 7 [*p* < 0.0001]; *I*^2^ = 96%) (Figure 9). Furthermore, meta-regression analysis was conducted using “type of therapy” as an ordinal variable, there were no differences between acupuncture and acupoint sticking (Supplementary Figure 15).

### PDSS scores

**Figure 10:**
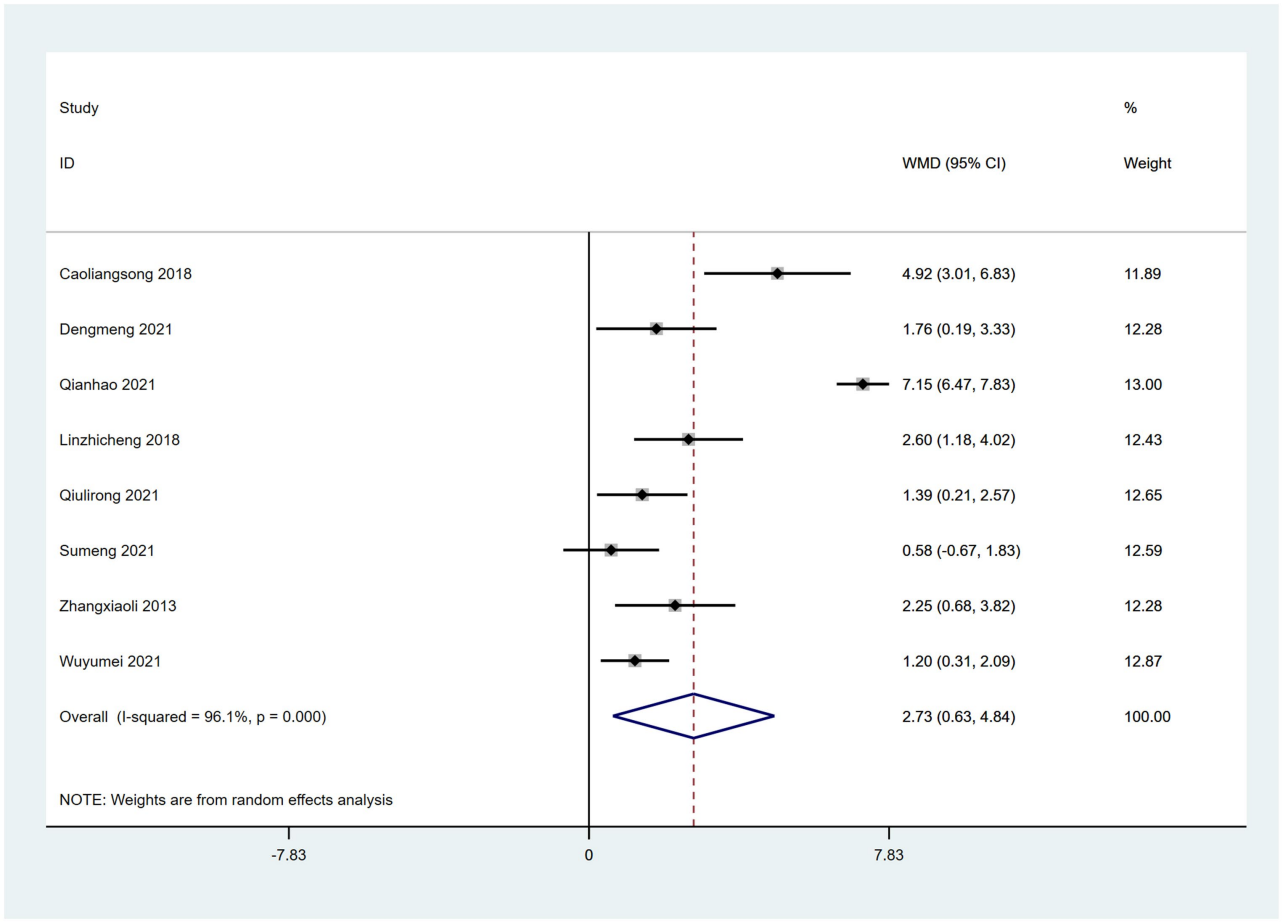
Forest plot comparing the effects of acupoint therapies with drugs versus drugs alone on PDSS.

Nine RCTs (44, 59, 60, 62, 64, 66, 82, 83, 85) reported the use of PDSS, which assesses cognitive function. PDSS scores were significantly better in patients treated with a combination of acupoint therapy with drugs than in those treated with drugs alone. A random effects model was used for the analysis (WMD = 15.87; 95% CI, 6.27 to 25.47; *Z* = 3.24; *p* = 0.001). Heterogeneity was observed among studies (chi-squared = 170.54; *df* = 8 [*p* < 0.0001]; *I*^2^ = 95.3%) (Figure 10). Meta-regression analysis using “type of therapy” as an ordinal variable was conducted, there were no differences between acupuncture and acupoint sticking (Supplementary Figure 16). The funnel plot test for publication bias is shown (Supplementary Figure 17). Egger’s bias test (*p* > 0.05) indicated no publication bias (Supplementary Figure 18).

### PSQI scores

**Figure 11:**
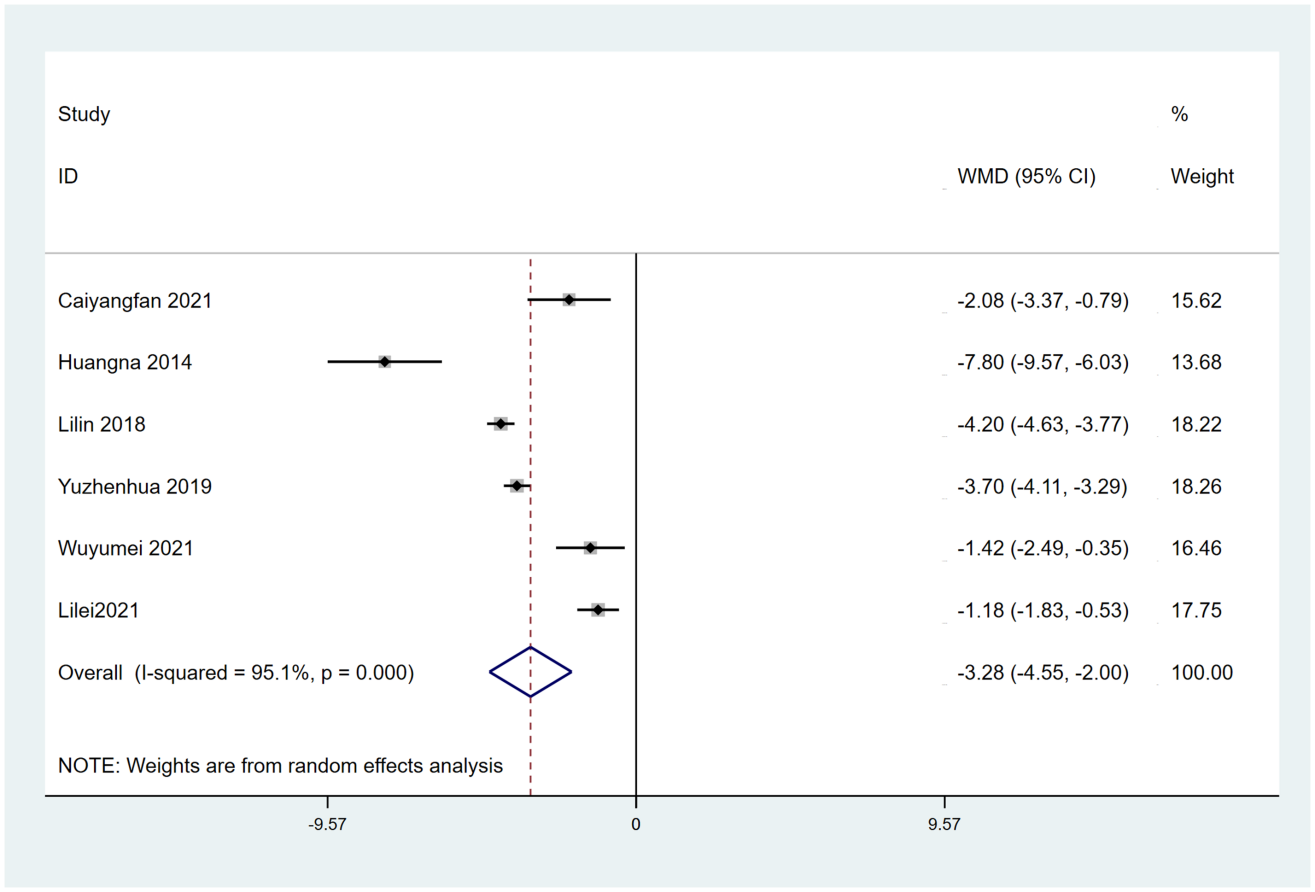
Forest plot comparing the effects of acupoint therapies with other therapies versus other therapies alone on PSQI.

Six RCTs reported the use of PSQI, which assesses sleep quality. A random effects model revealed that sleep quality was significantly better in patients treated with a combination of acupoint therapy with other therapies than in those treated with other therapies alone (WMD = −3.28; 95% CI, −4.55 to −2.00; *Z* = 5.03; *p* < 0.00001). Heterogeneity was observed among studies (chi-squared = 101.91; *df* = 5 [*p* < 0.0001]; *I*^2^ = 95%) (Figure 11). The results of the sensitivity analysis were not significantly different from the aforementioned results. When meta-regression analysis was conducted used “type of therapy” as an ordinal variable, the result showed no difference between acupuncture and acupoint sticking (Supplementary Figure 19). The funnel plot test for publication bias is shown (Supplementary Figure 20). Egger’s bias test indicated no publication bias (Supplementary Figure 21).

### PAC-QoL scores

**Figure 12:**
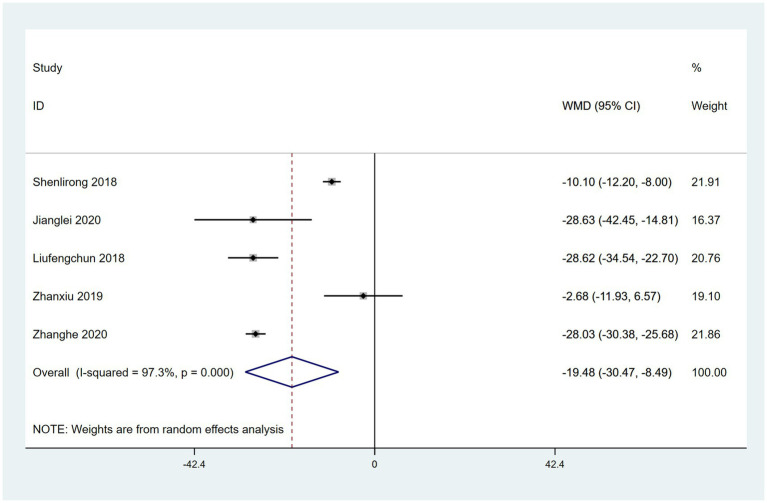
Forest plot comparing the effects of acupoint therapies with other therapies versus other therapies alone on PAC-QoL scores.

Five RCTs reported the use of PAC-QoL, which assesses quality of life. A random effects model revealed that PAC-QoL was significantly better in patients treated with a combination of acupoint therapy with other therapies than in those treated with other therapies alone (WMD = −19.48; 95% CI, −30.47 to −8.49; *Z* = 3.47; *p* = 0.0005). Heterogeneity was observed among studies (chi-squared = 149.55; *df* = 4 [*p* < 0.00001]; *I*^2^ = 97.3%) (Figure 12). Meta-regression analysis using “type of therapy” as an ordinal variable,” showed there were no differences between treatments (Supplementary Figure 22). The results of the sensitivity analysis were not significantly different from the aforementioned results. Egger’s bias test indicated no publication bias (Supplementary Figure 23). The results of the meta-analysis for all outcome indicators were summarized in Supplementary Table 3.

### Adverse events

Safety conditions were reported in 12 RCTs (44, 64, 65, 66, 69, 71, 74, 75, 78, 79, 82, 85) and adverse effects were not mentioned in 30 studies. Of the 12 studies that mentioned adverse effects, seven trials (21, 65, 69, 70, 78, 79, 82) reported a negative association with acupoint therapies; the other five trials (64, 66, 71, 75, 85) reported no such association. The observed adverse effects were mainly gastrointestinal symptoms (such as discomfort, vomiting, and nausea), subcutaneous hematoma, headache, or were related to the insertion of needles. All of these adverse effects were mild and showed signs of reversibility.

## Discussion

Medication is the main treatment for PD, but its long-term use has many potential complications. Dopaminergic treatments also have limitations in the treatment of PD-NMS (86). In the present study, we reviewed the feasibility, efficacy, and safety of acupoint therapies for managing PD-NMS using 42 included studies. To the best of our knowledge, this is the first meta-analysis to address this specific question. Our preliminary data indicated the feasibility of acupoint therapies for PD-NMS without serious adverse events. The meta-analysis revealed that acupoint therapy combined with CM was superior to CM alone in terms of the total effective rate, as well as HAMD, MMSE, MoCA, PDSS, PSQI, and PAC-QoL scores. However, only two included studies measured MMSE.

One previous review (87) reported that acupoint therapy is a helpful intervention for relieving the motor symptoms of PD, and another indicated that acupuncture can relieve PD-NMS (88). To date, however, there have been no reviews of acupoint therapy other than acupuncture for PD-NMS treatment. Furthermore, the evidence for acupoint therapy in the management of PD-NMS remains inconclusive because of the limited availability of clinical trials, often with small sample sizes and suboptimal methodological quality (87–89). Compared with previous reviews, the present study used a more comprehensive search by including more recently published trials. Although evidence for the use of acupoint therapy to alleviate PD-NMS was mixed in the current study, likely because of high heterogeneity in participant characteristics and study designs, some interesting observations were noted. First, thirteenth studies used HAMD to evaluate anxiety and depression, nine studies used PDSS and six studies used PSQI to evaluate sleep quality, two studies used MMSE and eight studies used MoCA to evaluate intellectual status and cognitive function. This finding indicates that sleep disturbances, anxiety, depression, and reduced cognitive function are common in patients with PD. Second, only one study (44) evaluated acupoint sticking therapy in terms of PDSS scores, and one study evaluated this therapy in terms of PAC-QoL scores (50). These findings indicate that acupoint sticking for the treatment of PD-NMS is relatively uncommon, and conclusive conclusions cannot be made about its efficacy and safety. Further exploration is therefore required to explore whether PD-NMS patients can benefit from acupoint sticking therapy.

In terms of effective rates, our meta-analysis results indicated that acupoint therapy combined with CM or NDT showed more benefits than treatment with CM or NDT alone. Effective rates is the percentage of improvement in the patient’s condition with therapy. Studies using this indicator are based on TCM clinical symptom improvement criteria. In other previous studies, acupoint therapy combined with CM or NDT was also reportedly more effective, consistent with our findings (49–51, 53–58, 60, 61, 63–65, 68, 70, 71, 73–76, 78, 81–85). In addition, our subgroup analysis based on intervention type indicated that a combination of acupoint therapy plus western drugs was significantly better than western drugs alone, and that a combination of acupoint therapy plus other therapies was better than other therapies alone. Also, we performed subgroup analysis according to the treatment method in control group and the heterogeneity still remained high. We considered that this might be due to the large number of included studies and the relative diversity of acupoint selection or treatment method in control group. In conclusion, acupoint therapy may be applicable for the treatment of PD-NMS.

Depression is a common feature in PD, with an estimated prevalence of 30–50% (90). HAMD is most commonly applied to evaluate depression (96). Our findings revealed that acupoint therapy combined with CM or NDT resulted in a significant improvement in depression compared with CM or NDT alone. This finding is consistent with some previous studies (59, 65, 66, 73, 74, 76, 78, 79, 81, 84). Moreover, a previous qualitative review focusing on PD-NMS reported that acupuncture likely has a positive effect on PD-related depression (91). Our results showed the heterogeneity still remained high in these researches of acupoint therapy with drugs versus drugs alone after conducting subgroup analysis, though there was no heterogeneity in these researches of acupoint therapy combined with other therapies. We considered that this might be due to the large number of included studies and the relative diversity of acupoint selection or treatment method in control group. Although acupoint therapy was able to effectively ameliorate PD-related depressive symptoms, more evidence is needed because the sensitivity analysis suggested relative instability in the results.

In terms of cognition, we found evidence of an overall greater effect of acupoint therapy combined with CM than CM alone. Eight studies (45, 59, 68, 69, 71, 75, 77, 80) reported that acupoint therapy combined with CM improved MoCA scores more than CM alone. And we hypothesized that the high heterogeneity was due to the diversity of treatment method and duration differences. Two studies (45, 46) demonstrated that acupuncture combined with CM improved MMSE scores more than CM alone, but the effect was not significant. We speculate that the discrepancy between MMSE scores and MoCA scores may be attributed to the fact that only two studies using MMSE scores as an outcome measure were retrieved, which showed significant heterogeneity. Therefore, more related research is needed to support this finding. Other studies have also indicated that acupuncture exerts a positive effect on global cognitive function (92, 93).

In terms of PD-related sleep quality, nine RCTs (44, 59, 60, 62, 64, 66, 82, 83, 85) reported the use of PDSS for assessing sleep quality. PDSS scores were significantly better in patients treated with a combination of acupoint therapy with drugs than in those treated with drugs alone. However, the heterogeneity was high. We reviewed the nine studies and concluded that the reason may be the diversity of acupoint selection and the difference of treatment course. Six RCTs reported the use of PSQI for assessing sleep quality. Similar to the PDSS results, PSQI scores were significantly better in patients treated with a combination of acupoint therapy with CM or other therapies than in those treated with CM or other therapies alone. The high heterogeneity may be due to the diversity of acupoint therapy method or treatment method in control group. In addition, acupuncture is reportedly effective for various types of insomnia (94). However, the effects of types of acupoint therapy other than acupuncture on this PD-NMS need to be further studied and discussed.

Constipation is a predominant early PD-NMS, and some evidence suggests that the initial pathology of PD arises inside the central nervous system (95). Our results regarding PAC-QoL revealed that the effects of acupoint therapy combined with CM for PD-related constipation were better than with CM alone. The high heterogeneity may be due to the diversity of treatment method in control group or the difference of treatment course. Although acupuncture is considered an effective and safe treatment for constipation in patients without PD on the basis of previous randomized trials (95, 96), more high-quality evidence-based studies are needed to confirm the effectiveness of acupoint therapy on PD-related constipation.

Our study showed acupoint therapy combined with CM or NDT yielded superior results. According to meridian and acupoints theory in TCM, acupoint therapy regulates Qi (energy) and blood and maintains the Yin and Yang (two energy subdivisions) balance of internal organs by stimulating acupoints corresponding to symptomatic organs or body areas, thereby relieving symptoms. Empirical studies have found that acupoint therapy improves PD-NMS by modulating the levels of 5-hydroxytryptamine (5-HT), dopamine (DA) and brain-derived neurotrophic factor (BDNF) in serum (77, 84).

In the aforementioned results, almost all outcome indicators showed large heterogeneity. This may be attributable to the following reasons: (i) the relatively small sample sizes of the included articles; (ii) differences in the professional levels of acupuncturists or practitioners, the selection of acupuncture points, and/or the frequency of therapies; (iii) the different initial severity levels of patients included in the trials, which may have resulted in clinical heterogeneity between studies; and (iv) differences in the duration of each acupoint therapy and the intervention intensity across all studies. In addition, most studies had a short intervention time, meaning that their results only reflect the effects of short-term acupoint therapy for PD-NMS. Therefore, there is a need for large-sample, multicenter, long-term RCTs in the future. PD is a chronic progressive disease. At present, the challenge is not only to relieve symptoms but also to delay the progression of the disease. Therefore, the long-term effects of acupoint therapy in treating PD-NMS should also be taken into consideration. However, the present review highlights the possible therapeutic effectiveness of acupuncture therapies for PD-NMS.

### Limitations and strengths

To our knowledge, our study is the first to comprehensively examine acupoint therapy for PD-NMS, although some symptoms (e.g., dizziness, bladder urgency, and apathy) were not reviewed because of a lack of original data. Our study combined data from 42 eligible studies that covered an array of common PD-NMS; our findings provide potential evidence for several non-pharmaceutical therapies for PD-NMS. Previous studies have mostly focused on motor symptoms (97, 98) or have reviewed the effects of complementary therapies (including acupuncture) for motor and non-motor symptoms but did not provide a quantitative analysis (99). Therefore, our study represents a comprehensive assessment of the currently available evidence regarding PD-NMS.

Given that acupoint therapy is a manipulated intervention, blinding for practitioners is hard to implement in most studies (even in studies outside of PD) (100). Consequently, concerns about the risk of bias in our study mostly existed in the domains of blinding—particularly in participant and personnel blinding—and weakened the strength of the evidence, and thus of our conclusions. Furthermore, in the present study, we included acupoint sticking therapy as an intervention method, and confirmed that acupoint sticking combined with conventional care was more effective for PD-NMS than conventional care alone. Some of the pooled results also continued to exhibit high levels of heterogeneity and were unstable in the sensitivity analyses, despite restricting the criteria for study enrollment and performing subgroup analyses. Another major limitation of the study was that all trials were conducted in China; this may affect the generalizability of our findings. In addition, there were no studies on Guasha, cupping, or acupressure interventions in PD-NMS, so we were unable to assess the efficacy of these treatments for PD-NMS. Our findings on the effectiveness of acupoint therapy in PD-NMS should therefore be interpreted with caution.

### Implications for clinical practice

Some results from the present study may provide guidance for clinical applications. First, for patients with PD-related depression or decreased quality of life or cognition, acupoint therapy may be a good choice. Second, acupoint therapy combined with CM may be suitable for treating PD-related depression, cognitive function, insomnia, and constipation. Third, acupoint sticking therapy may be used to treat constipation.

## Conclusion

The results of the present meta-analysis indicate that acupoint therapy may be associated with improvements in various PD-NMS including depression, intellectual status/cognitive function, sleep quality, and constipation.

## Data Availability

The original contributions presented in the study are included in the article/Supplementary material, further inquiries can be directed to the corresponding author.
