# Supplementary material for: Combination treatment with acupoint therapy and conventional medication for non-motor symptoms in Parkinson’s disease: a systematic review and meta-analysis
Source: Front Neurol. 2025 May 22;16:1381500. doi: 10.3389/fneur.2025.1381500 (PMC12137075; doi:10.3389/fneur.2025.1381500)
Supplement: Supplementary file 1 [file Data_Sheet_1.docx]

Table 1. Summary of basic characteristics of the included studies.

| NO. | First author (year) | Sample size (proportion of female) (n/%) | | Age in years Mean (SD) | | Randomization  method | Treatment | Control | Frequency | Location | Treatment  duration | Outcome | Safety |
| --- | --- | --- | --- | --- | --- | --- | --- | --- | --- | --- | --- | --- | --- |
|  |  | Exp | Con | Exp | Con |  |  |  |  |  |  |  |  |
| 1 | Caiyangfan 2021 | 25 (10/40%) | 25 (8/32%） | 69.32(7.87) | 70.24 (7.77) | Random  number  table | Acupoint sticking + tolterodine tartrate tablets | tolterodine tartrate tablets | 5 times every week | Fujian | 5 w | PDSS;PSQI | Not reported |
| 2 | Caoliangsong 2018 | 48(20/41.67%） | 48(19/39.58%） | 56.31(9.62) | 55.31(9.42) | Random  number  table | Acupuncture + cognitive function training | cognitive function training | Per day | Hubei | 42d | MMSE;MOCA | Not reported |
| 3 | Chaiqing 2022 | 39（17/43.59%） | 39（19/48.72%) | 74.52(3.1) | 75.02 (4.65) | Random  number  table | Acupuncture + Bifidobacterium + compound levodopa | Bifidobacterium + compound levodopa | 5 times every week | Tianjin | 12 w | MMSE | Not reported |
| 4 | Shenlirong 2018 | 30（12/40%） | 30（13/43.33%) | 74(11) | 72(11) | Random  number  table | Acupuncture + Maren Capsule | Maren Capsule | One time every two days | Shanghai | 4 w | PAC-QoL | Not reported |
| 5 | Jianglei 2020 | 33(15/45.45%） | 33（16/48.48%) | 64.82(12.23) | 64.41(11.89) | Random  number  table | Acupuncture + Levodopa and Benserazide Hydrochloride Tablets | Levodopa and Benserazide Hydrochloride Tablets | Per day | Hubei | 20 d | PAC-QoL | Not reported |
| 6 | Liuaiguo 2018 | 48(20/41.67%） | 48（23/47.92%) | 65.28(7.32) | 64.58(7.33) | Random  number  table | Acupuncture + Compound Carbidopa Tablets | Compound Carbidopa Tablets | Per day | Hebei | 7 d | Effective rate | Not reported |
| 7 | Liufengchun 2018 | 60(27/45%） | 60（24/40%) | 64.67(10.47) | 63.13(8.63) | Convenience Sampling | acupoint sticking + conventional diet care | conventional diet care | Per day | Beijing | 15 d | Effective rate; PAC-QoL | Not reported |
| 8 | Lvjindan 2014 | 30（14/46.67%） | 30（13/43.33%) | 68.07(5.24) | 65.53(11.23) | Random grouping | Warm Parcle + conventional diet care | Warm Parcle | 2 times every day | Guangdong | 20 d | Effective rate | Not reported |
| 9 | Luosining 2019 | 30(12/40%） | 30（13/43.33%) | 66.31(5.57) | 66.07(5.48) | Random  number  table | Acupuncture + Phenolphthalein Tablets | Phenolphthalein Tablets | Per day | Shanxi | 4 w | Effective rate | Not reported |
| 10 | Xuexiaoli 2018 | 50（20/40%） | 50（18/36%) | 60.17(9.25) | 62.13(8.04) | Random  number  table | Acupuncture and moxibustion（Wenzhenjiu） and Simo Soup（Simotang | Simo Soup（Simotang | Per day | Jiangsu | 7 d | Effective rate | Not reported |
| 11 | Zhanxiu 2019 | 20（12/60%） | 20（9/45%) | 61.87(6.25) | 63.14(7.28) | Random  number  table | Acupuncture + Maren pill | Maren pill | 6 times every week | Anhui | 8 w | Effective rate; PAC-QoL | Not reported |
| 12 | Zhanghe 2020 | 34(19/55.88%） | 34（16/47.06%) | 65.29(7.79) | 65.62(8.09) | Random grouping | Abdominal needle and Maren capsule | Maren capsule | 3 times per week | Guangdong | 4 w | Effective rate; PAC-QoL | Not reported |
| 13 | Caili 2017 | 29（12/41.38%） | 29（11/37.93%) | 56.84(3.12) | 58.25(2.84) | Random grouping | Acupuncture and Zhichan soup | Zhichan soup | Per day | Shanghai | 4 w | Effective rate | Not reported |
| 14 | Chenhongbao 2021 | 31（13/41.94%） | 31（15/48.39%) | 74.32.(5.12) | 74.54(5.03) | Random grouping | Acupuncture and Guipi Tang | Guipi Tang | 1 time every two days | Chongqing | 14 d | Effective rate | Not reported |
| 15 | Daijunfeng 2019 | 52(21/40.38%） | 52（19/36.54%) | 61.02(3.57) | 60.97(3.54) | Random grouping | Acupuncture and repeated transcranial magnetic stimulation (rTMS) | repeated transcranial magnetic stimulation(rTMS) | Per day | Henan | 2 w | Effective rate | Not reported |
| 16 | Dengmeng 2021 | 53（26/49.06%） | 53（24/45.28%) | 65.12(5.1) | 65.61(5.35) | Random  number  table | Acupuncture and Dingchananshen soup | Dingchananshen soup | 5 times every week | Hubei | 4 w | HAMD; MOCA; PDSS | Not reported |
| 17 | Dongqinjian 2018 | 36（17/47.22%） | 36（15/41.67%) | 60.25(6.376) | 59.06(7.83) | Random grouping | Acupuncture + Levodopa and Benserazide Hydrochloride Tablets | Levodopa and Benserazide Hydrochloride Tablets | Per day | Sichuan | 30 d | Effective rate;PDSS | Not reported |
| 18 | Haodongsheng 2018 | 58（22/37.93%） | 58（20/34.48) | 59.03(8.67) | 58.67(8.59) | Random  number  table | Acupuncture and repeated transcranial magnetic stimulation (rTMS) | rTMS | Per day | Henan | 2 w | Effective rate ;HAMD | Not reported |
| 19 | Huwanqin 2021 | 30（10/33.3%） | 30（14/46.67%) | 73.07(2.17) | 73.03(2.19） | Random  number  table | Acupuncture + Levodopa and Benserazide Hydrochloride Tablets | Levodopa and Benserazide Hydrochloride Tablets | 6 times every week | Guizhou | 4 w | PDSS | Not reported |
| 20 | Huangna 2014 | 20（7/35%） | 20（8/40%) | 61(8) | 59(9) | Order of treatment | Acupuncture + Levodopa and Benserazide Hydrochloride Tablets | Acupuncture + Levodopa and Benserazide Hydrochloride Tablets | 5 times every week | Shanxi | 4 w | Effective rate ;PSQI | Not reported |
| 21 | Huangyanxi 2020 | 34(15/44.12%） | 33（15/45.45%) | 58.47(6.75) | 59.24(6.89) | Random distribution of numbers | needle warming moxibustion + western medicine | western medicine | 3 times every week | Fujian | 12 w | Effective rate; PDSS | Y |
| 22 | Liulifang 2020 | 30（-/-） | 30（-/-) | 59.31(13) | 54.55(1.21) | Random allocation | Acupuncture + Duloxetine Hydrochloride Enteric-coated Tablets | Duloxetine Hydrochloride Enteric-coated Tablets | Per day | Guangdong | 28 d | Effective rate; HAMD | Y |
| 23 | Zhangyujuan 2022 | 43(23/53.49%） | 43（22/51.16%) | 61.8(4.6) | 62.2(4.3) | Random  number  table | Acupuncture + Levodopa and Benserazide Hydrochloride Tablets | Levodopa and Benserazide Hydrochloride Tablets | 4 times every week | Shandong | 8 w | HAMD;PDSS | Y |
| 24 | Lilin 2018 | 25(12/48%） | 25（12/48%) | 69.6(8.9) | 9.7(8.6) | Computer randomization | Acupuncture + Guipi soup | Guipi soup | Per day | Liaoning | 60 d | PSQI | Not reported |
| 25 | Qianhao 2021 | 52（26/50%） | 52（24/46.15%) | 60.45(2.40) | 60.33(2.37) | Random allocation | Acupuncture + Levodopa and Benserazide Hydrochloride Tablets | Levodopa and Benserazide Hydrochloride Tablets | 6 times every week | Guangdong | 4 w | Effective rate; MOCA | Not reported |
| 26 | Linzhicheng 2018 | 19（7/36.84%） | 18（5/27.78%) | 58.3(4.6) | 59.2(4.4) | Random  number  table | acupuncture + Donepezil) | Donepezil | 6 times every week | Fujian | 6 w | MOCA | Y |
| 27 | Luweijing 2022 | 32（15/46.88%） | 32（17/）53.12% | 61.37(1.61) | 63.78(1.31) | Random  number  table | Acupuncture + antiparkinsonian | antiparkinsonian | 4 times every week | Guangdong | 4 w | Effective rate | Y |
| 28 | Qiulirong 2021 | 26（11/42.31%） | 26（13/50%) | 61.19(5.98) | 61.23(6.51) | Random  number  table | acupuncture + Donepezil hydrochloride tablets | Donepezil hydrochloride tablets | 6 times every week | Shandong | 8 w | MOCA | Y |
| 29 | Yuzhenhua 2019 | 30（15/50%） | 30（15/50%) | 65.9(6.9) | 66.3(5.6) | Computer randomization | acupuncture + Dihuang Decoction+Levodopa | Dihuang Decoction+Levodopa | Per day | Beijing | 40 d | PSQI | Not reported |
| 30 | Xiayi 2012 | 30（8/26.67%） | 30（10/33.33%) | 72(7) | 72(8) | Order of treatment | acupuncture + Levodopa and Benserazide Hydrochloride Tablets | Levodopa and Benserazide Hydrochloride Tablets | 4 times every week | Jiangsu | 90 d | Effective rate; HAMD | Not reported |
| 31 | Luzhou 2022 | 32（16/50%） | 33（15/45.45%) | 67.81(7.27) | 67.36(8.83) | Random  number  table | acupuncture + Levodopa and Benserazide Hydrochloride Tablets | Levodopa and Benserazide Hydrochloride Tablets | 3 times every week | Shanghai | 8 w | Effective rate; HAMD | Not reported |
| 32 | Sumeng 2021 | 26（11/42，31%） | 27（12/44.44%) | 63.92(4.33) | 64.30(4.43) | Random  number  table | acupuncture + Levodopa and Benserazide Hydrochloride Tablets | Levodopa and Benserazide Hydrochloride Tablets | 6 times every week | Shandong | 8 w | Effective rate; MOCA | Y |
| 33 | Tianyu 2016 | 50（9/18%） | 50（12/24%) | 62.4(5.11) | 60.8(4.4) | Random  number  table | acupuncture + Levodopa and Benserazide Hydrochloride Tablets | Levodopa and Benserazide Hydrochloride Tablets | 1 time every 3 days | Neimenggu | 3 m | Effective rate; HAMD | Not reported |
| 34 | Zhangxiaoli 2013 | 24（8/33.33%） | 24（10/41.67%) | 67(6) | 66(5) | Random  number  table | acupuncture + Levodopa and Benserazide Hydrochloride Tablets+Tianmasu | Levodopa and Benserazide Hydrochloride Tablets+Tianmasu | 6 times every week | Jiangsu | 8 w | MOCA | Not reported |
| 35 | Gujing 2023 | 52（24/46.15%） | 51（21/41.18%) | 51.37(7.26) | 52.07(7.56) | Random  number  table | acupuncture + Levodopa and Benserazide Hydrochloride +Sertraline Hydrochloride Tablets | Levodopa and Benserazide Hydrochloride +Sertraline Hydrochloride Tablets | 5 times every week | Shanghai | 3 m | Effective rate; HAMD | Y |
| 36 | Wangxiaomei 2020 | 40(24/60%） | 40（22/55%) | 64.2(7.8) | 65.9(8) | Random  number  table | Heat sensitive moxibustion + levodopa hydrochloride benzselazy tablets and Paxil tablets | levodopa hydrochloride benzselazy tablets and Paxil tablets | Per day | Zhejiang | 2 m | HAMD | Y |
| 37 | Wuyumei 2021 | 31（21/67.71%） | 31（19/61.29%) | 66.65(9.66) | 66.23(10.47) | Random  number  table | Acupoint sticking + routine nursing | routine nursing | 5 times every week | Guangdong | 7 d | HAMD; MOCA; PSQI | Not reported |
| 38 | Yuanying 2020 | 46（15/31.61%） | 51（23/45.10%) | 75.5(8.7) | 63.7(7.9) | Random grouping | acupuncture + Escitalopram tablet | Escitalopram tablet | 4 times every week | Beijing | 3 m | Effective rate; HAMD | Not reported |
| 39 | Zhouchen 2016 | 63（33/52.38%） | 64（32/50%) | 69.2(4.6) | 70.1(4.4) | Random  number  table | acupuncture + Levodopa and Benserazide Hydrochloride Tablets | Levodopa and Benserazide Hydrochloride Tablets | Per day | Guangdong | 8 w | Effective rate; PDSS | Y |
| 40 | Lilei 2021 | 50（23/46%）; | 50（24/48%) | 61.56(7.51) | 62.49(7.53) | Random  number  table | acupuncture + Levodopa and Benserazide Hydrochloride Tablets | Levodopa and Benserazide Hydrochloride Tablets | Per day | Henan | 8 w | Effective rate; PDSS | Not reported |
| 41 | Zhaoyanping 2019 | 32（15/46.88%） | 31（15/48.39%) | 61.56(7.51) | 64.85(6.14) | Random  number  table | acupuncture + acupuncture + | Paxil tablets | 6 times every week | Anhui | 8 w | Effective rate; HAMD | Not reported |
| 42 | Xuyiwei 2020 | 33（18/55.55%） | 37（16/43.24%) | 61.736(10.28) | 61.95(9.77) | Envelope grouping | acupuncture + Levodopa and Benserazide Hydrochloride Tablets | Levodopa and Benserazide Hydrochloride Tablets | 4 times every week | Guangdong | 2 w | Effective rate;PDSS | Y |

PDSS: Parkinson's Disease Sleep Scale; PSQI: Pittsburgh Sleep Quality Index; HAMD: Hamilton Depression Rating Scale; MOCA: Montreal Cognitive Assessment; MMSE: Mini-mental State Examination; PAC-QoL: Patient-Assessment of Constipation Quality Of Life; Exp: Experimental group; Con: Control group; w: week; d:day; m:month; Y: Yes;

None of the studies reported the blinding status.

Table 2. Summary of basic characteristics of the patients of included studies.

| NO. | First author (year) | Sample size (Proportion of Female) | | Disease duration(Exp/Con) | HAMD | MMSE | PAC-QoL | MOCA | PDSS | PSQI | PAC-QoL | Drop out rate |
| --- | --- | --- | --- | --- | --- | --- | --- | --- | --- | --- | --- | --- |
|  |  | Exp | Con |  |  |  |  |  |  |  |  |  |
| 1 | Caiyangfan 2021 | 25 (10/40%) | 25 (8/32%） | 4..14±2.67/4.10±2.62 | / | / | / | / | 95.04 ±16. 13/95.68 ±16.29 | 14.76±1.54/14.88±2.22 | / | None |
| 2 | Caoliangsong 2018 | 48(20/41.67%） | 48(19/39.58%） | 3.73± 2.09/3.62 ±1.53 | / | 17.23± 3.14/17.46 ±3.27 | / | 18.66± 3.17/18.42 ±2.96 | / | / | / | None |
| 3 | Chaiqing 2022 | 39（17/43.59%） | 39（19/48.72%) | 3.63± 1.03/3.32±1.61 | / | 26.51±2.40/25.77±3. 19 | / | / | / | / | / | None |
| 4 | Shenlirong 2018 | 30（12/40%） | 30（13/43.33%) | 8.53±1.55  /7.13±1.47 | / | / | / | / | / | / | / | None |
| 5 | Jianglei 2020 | 33(15/45.45%） | 33（16/48.48%) | 3.76±1.76/3.79±1.86 | / | / | / | / | / | / | 78.21±22.36/76.18±24.32 |  |
| 6 | Liuaiguo 2018 | 48(20/41.67%） | 48（23/47.92%) | 5.17±2.35/5.27±2.54 | / | / | / | / | / | / | / | None |
| 7 | Liufengchun 2018 | 60(27/45%） | 60（24/40%) | 6.47±10.47/4.65±3.08 | / | / | 81.92±14.14/80.75±1.24 | / | / | / | / | None |
| 8 | Lvjindan 2014 | 30（14/46.67%） | 30（13/43.33%) | 5.08±2.96/4.68±2.88 | / | / |  | / | / | / | / | None |
| 9 | Luosining 2019 | 30(12/40%） | 30（13/43.33%) | Not mentioned | / | / | / | / | / | / | / | None |
| 10 | Xuexiaoli 2018 | 50（20/40%） | 50（18/36%) | 3.92±1.29  /4.02±1.33 | / | / | / | / | / | / | / | None |
| 11 | Zhanxiu 2019 | 20（12/60%） | 20（9/45%) | 5.89±4.62/5.754±3.17 | / | / | 78.82±19.43/79.95±20.19 | / | / | / | / | None |
| 12 | Zhanghe 2020 | 34(19/55.88%） | 34（16/47.06%) | 8.29±2.69/8.09±2.76 | / | / | 69.72±9.63 | / | / | / | / | None |
| 13 | Caili 2017 | 29（12/41.38%） | 29（11/37.93%) | 4.82±1.56/4.37±1.73 | / | / | 98.65±8.46  /97.46±7.95 | / | / | / | / | None |
| 14 | Chenhongbao 2021 | 31（13/41.94%） | 31（15/48.39%) | 4.32±1.21/4.27±1.19 | / | / | / | / | / | / | / | None |
| 15 | Daijunfeng 2019 | 52(21/40.38%） | 52（19/36.54%) | 2.74±1.12/2.71±1.15 | 14.92±2.76/14.89±2.74 | / | / | / | / | / | / | None |
| 16 | Dengmeng 2021 | 53（26/49.06%） | 53（24/45.28%) | 8.27±2.25/8.92±3.20 | / | 23.38±8.33  /22.51±8.16 | / | 22.51±4.16/22.38±4.33 | 112.27±28.51/112.69±28.32 | / | / | None |
| 17 | Dongqinjian 2018 | 36（17/47.22%） | 36（15/41.67%) | 4.03±0.878/3.94±1.013 | / | / | / | / | 110.53±8．853/112．08±12.046 | / | / | None |
| 18 | Haodongsheng 2018 | 58（22/37.93%） | 58（20/34.48) | 5.03±1.34  /4.37±1.25 | 14.75±2.61/ 14.46±2.57 | / | / | / | / | / | / | None |
| 19 | Huwanqin 2021 | 30（10/33.3%） | 30（14/46.67%) | Not mentioned | / | / | / | / | 54.73±11.34/50.37±10.94 | / | / | None |
| 20 | Huangna 2014 | 20（7/35%） | 20（8/40%) | 34±８/35±6 | / | / | / | / | / | 13.85±4.06/13.60±3.66 | / | None |
| 21 | Huangyanxi 2020 | 34(15/44.12%） | 33（15/45.45%) | 4.25±2.39/4.60±2.43 | / | / | / | / | 96.76±14.55/96.18±8.77 | / | / | 0/1 |
| 22 | Liulifang 2020 | 30（-/-） | 30（-/-) | 2.04±0.53/2.09±0.37 | 20.10 土2.22/20.82±2.18 | / | / | / | / | / | / | None |
| 23 | Zhangyujuan 2022 | 43(23/53.49%） | 43（22/51.16%) | 4.84± 1.53/4.58 ±1.68 | 14.51± 5.85/14.88±4.74 | / | / | / | 113.33± 18.79/110.56 ±19.53 | / | / | None |
| 24 | Lilin 2018 | 25(12/48%） | 25（12/48%) | Not mentioned | / | / | / | / | / | 14.9± 1. 24/15.1± 1.71 | / | None |
| 25 | Qianhao 2021 | 52（26/50%） | 52（24/46.15%) | 5.08±0.78/5.11±0.81 | / | / | / | 13.24±1.23/13.29±1.33 | / | / | / | None |
| 26 | Linzhicheng 2018 | 19（7/36.84%） | 18（5/27.78%) | 3.3±0.9/3.2±1.0 | / | / | / | 20.1±0.8/19.6±1.1 | / | / | / | None |
| 27 | Luweijing 2022 | 32（15/46，88%） | 32（17/）53.12% | 5(4,8.75)/4(1,8) | / | / | / | / | / | / | / | 3/3 |
| 28 | Qiulirong 2021 | 26（11/42.31%） | 26（13/50%) | 3±1.595/2.72±1.50 | / | / | / | 20.92±2.10/21.96±1.97 | / | / | / | 2/2 |
| 29 | Yuzhenhua 2019 | 30（15/50%） | 30（15/50%) | Not mentioned | / | / | / | / | / | 14.8±1.75/14.7±1.69 | / | None |
| 30 | Xiayi 2012 | 30（8/26.67%） | 30（10/33.33%) | 6.7±3.0/5.8±4.0 | 15.13±3.91/14.70±3.53 | / | / | / | / | / | / | None |
| 31 | Luzhou 2022 | 32（16/50%） | 33（15/45.45%) | 37.22±22.24/36.03±18.78 | 9.63±6.06/9.21±6.01 | / | / | / | / | / | / | 2/1 |
| 32 | Sumeng 2021 | 26（11/42，31%） | 27（12/44.44%) | 3.9±1.72/4.13±1.86 | / | / | / | 19.62±2.50/19.04±2.12 | / | / | / | 2/1 |
| 33 | Tianyu 2016 | 50（9/18%） | 50（12/24%) | 3.3±0.5/3.6±0.4 | 15.63±4.11/15.17±3.94 | / | / | / | / | / | / | None |
| 34 | Zhangxiaoli 2013 | 24（8/33.33%） | 24（10/41.67%) | 5.17±2.58/4.96±2.58 | / | / | / | 16.008±2.62/16.17±2.88 | / | / | / | None |
| 35 | Gujing 2023 | 52（24/46.15%） | 51（21/41.18%) | 4.67±0.82/4.21±0.79 | 15.69±2.34/15.27±2.11 | / | / | / | / | / | / | None |
| 36 | Wangxiaomei 2020 | 40(24/60%） | 40（22/55%) | 43.3±24.2/ 46.8±25.7 | 21.75±5.55/21.55±5.61 | / | / | / | / | / | / | None |
| 37 | Wuyumei 2021 | 31（21/67.71%） | 31（19/61.29%) | Not mentioned | 11.03±3.95 /10.90±4.23 | / | / | 25.71±2.88  /25.81±2.21 | / | 8.23±3.70/8.29±3.19 | / | None |
| 38 | Yuanying 2020 | 46（15/31.61%） | 51（23/45.10%) | 4.7±8.6/3.7±4.3 | 30.57±3.73/31.88±4.27 | / | / | / | / | / | / | None |
| 39 | Zhouchen 2016 | 63（33/52.38%） | 64（32/50%) | 7.43±3.85/（9.32±3.45 | / | / | / | / | 96.47±28.56/96.54±19.65 | / | / | None |
| 40 | Lilei 2021 | 50（23/46%）; | 50（24/48%) | 4.08±0.61/4.13±0.5 | / | / | / | 17.21 ±2.63/17.83±2.21 | 105.09 ±13.61/104.91 ±12.89 | / | / | None |
| 41 | Zhaoyanping 2019 | 32（15/46.88%） | 31（15/48.39%) | 4.5±2.7/4.2±2.9 | 21.15±6.21/20.37±5.72 | / | / | / | / | / | / | None |
| 42 | Xuyiwei 2020 | 33（18/55.55%） | 37（16/43.24) | 3.52 ± 2.78  /3.26 ± 2.32 | / | / | / | / | 113.39 ± 23.16  /112.78 ± 19.72 | / | / | 7.8 % (6/76) |

Table 3. Summary of outcomes results of the included studies.

| Outcomes | Number of RCTs | Statistically significant difference | Heterogeneity | Measures that deal with greater heterogeneity | Significant difference after reducing heterogeneity | Publication bias |
| --- | --- | --- | --- | --- | --- | --- |
|  |  |  |  |  |  |  |
| Effective rates | 28 | Y | 71% | Subgroup analysis; removing articles that had a strong possibility of heterogeneity | *P* < 0.05 | N |
| HAMD | 13 | Y | 83.9% | Subgroup analysis; removing articles that had a strong possibility of heterogeneity | *P* < 0.00001 | N |
| MMSE | 2 | N | 93% | Random effects | *P* > 0.05 | Not applicable |
| MoCA | 8 | Y | 96% | Random effects | *P* < 0.00001 | N |
| PDSS | 9 | Y | 95.3% | Random effects | *P* < 0.0001 | N |
| PSQI | 6 | Y | 95% | Random effects | *P* < 0.00001 | N |
| PAC-QoL | 5 | Y | 97.3% | Random effects | *P* < 0.00001 | N |
