# Supplementary material for: Combination treatment with acupoint therapy and conventional medication for non-motor symptoms in Parkinson’s disease: a systematic review and meta-analysis
Source: Front Neurol. 2025 May 22;16:1381500. doi: 10.3389/fneur.2025.1381500 (PMC12137075; doi:10.3389/fneur.2025.1381500)
Supplement: Supplementary file 2 [file Data_Sheet_2.docx]

s Fig. 1 Forest plot comparing the effects of acupoint therapies plus western drugs versus western drugs alone on effective rates after removing four studies.


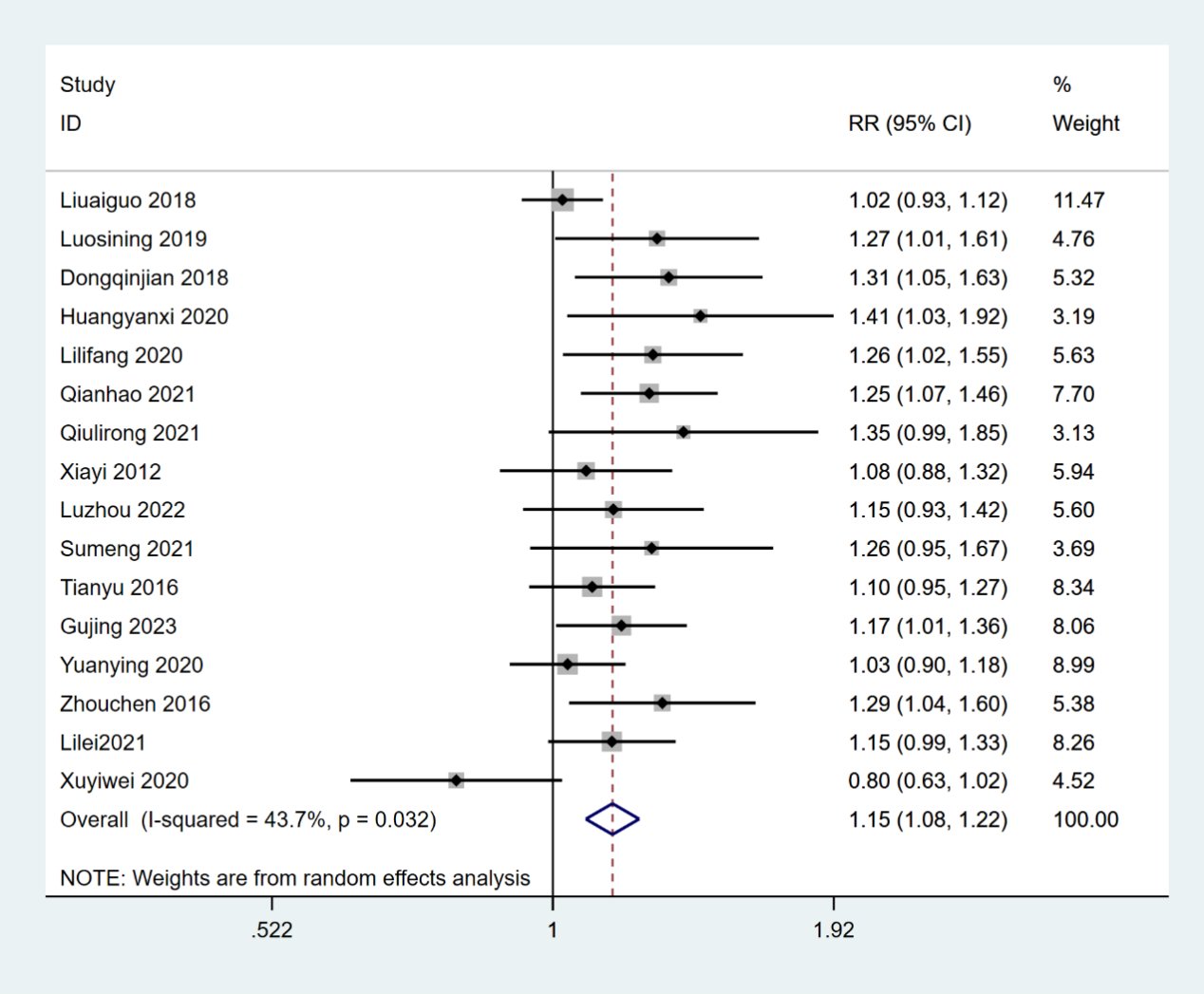


s Fig. 2 Funnel diagram comparing the effects of acupoint therapies plus western drugs versus western drugs alone on effective rates.


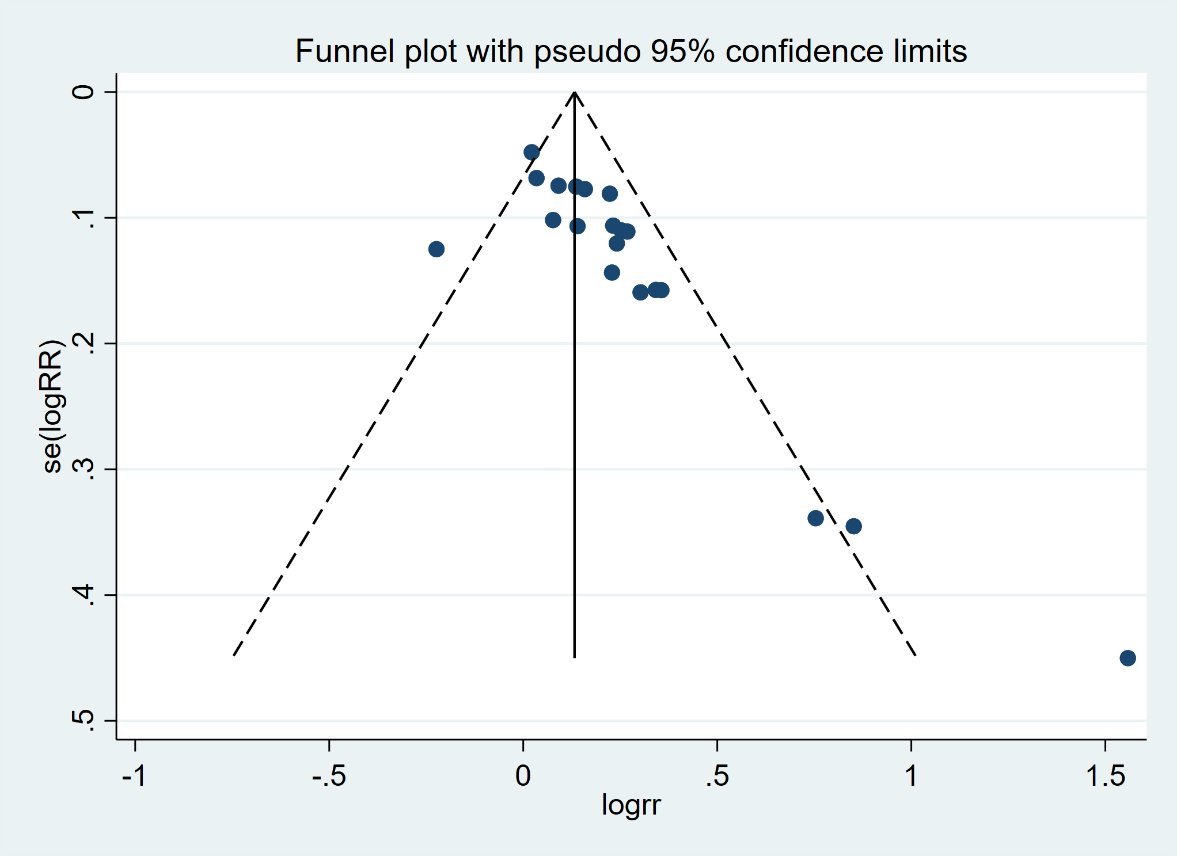


s Fig. 3 Egger’s bias test comparing the effects of acupoint therapies plus western drugs versus western drugs alone on effective rates.


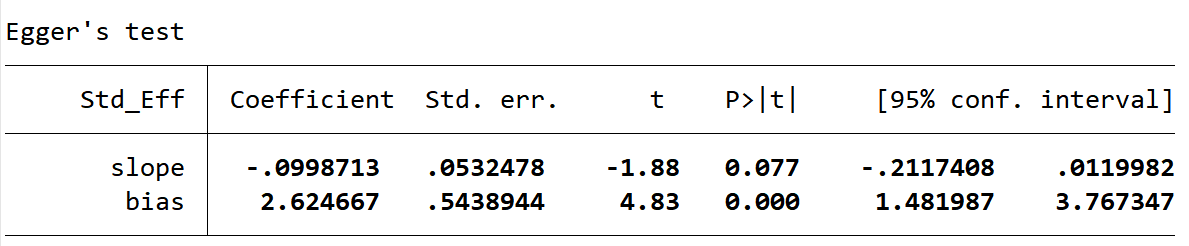


s Fig. 4 Comparison of the effects of acupoint therapies plus western drugs versus western drugs alone on effective rates after the shear compensation method.

**
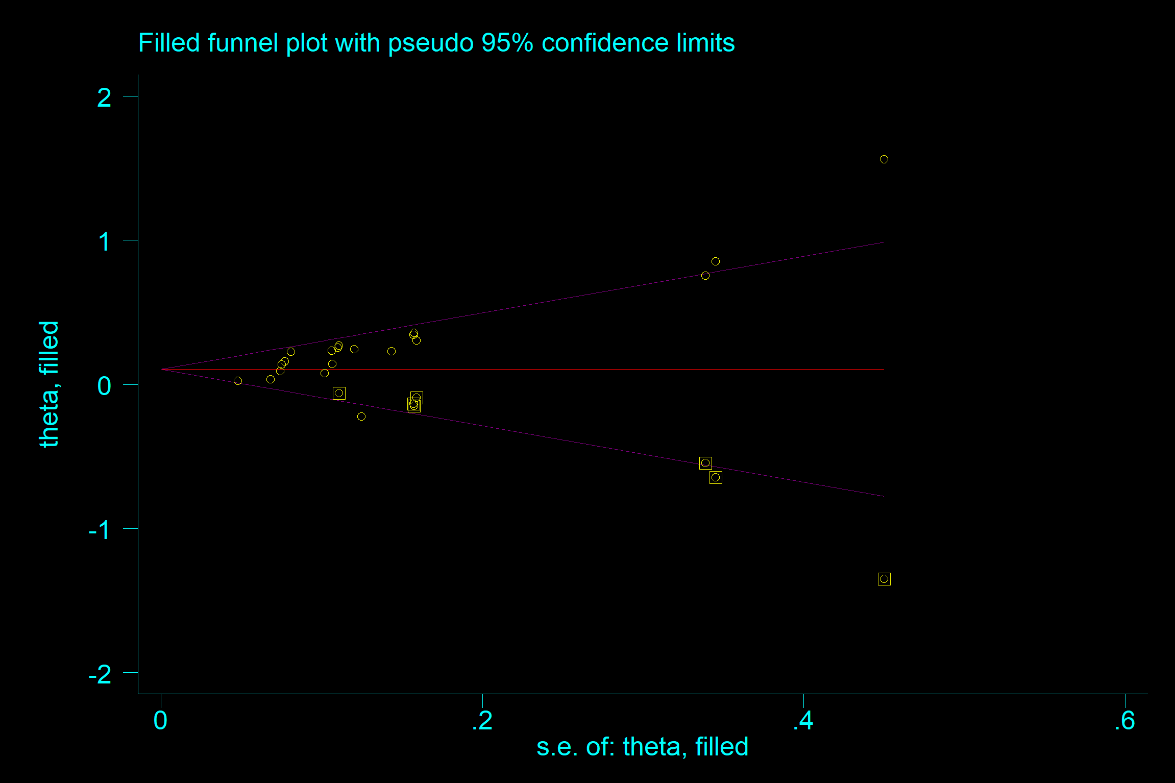
**

**Note：**s Fig. 1 shows a decrease in heterogeneity after the removal of four papers with a greater impact on heterogeneity. The funnel plot of 16 studies showed there was no publication bias (s Fig. 2). However, Egger’s bias test of 20 studies showed there was publication bias (s Fig. 3), so the shear compensation method was used to process the above results (s Fig. 4).

s Fig. 5 L’Abbe and Galbraith radial plots of acupoint therapies plus other therapies versus other therapies alone.


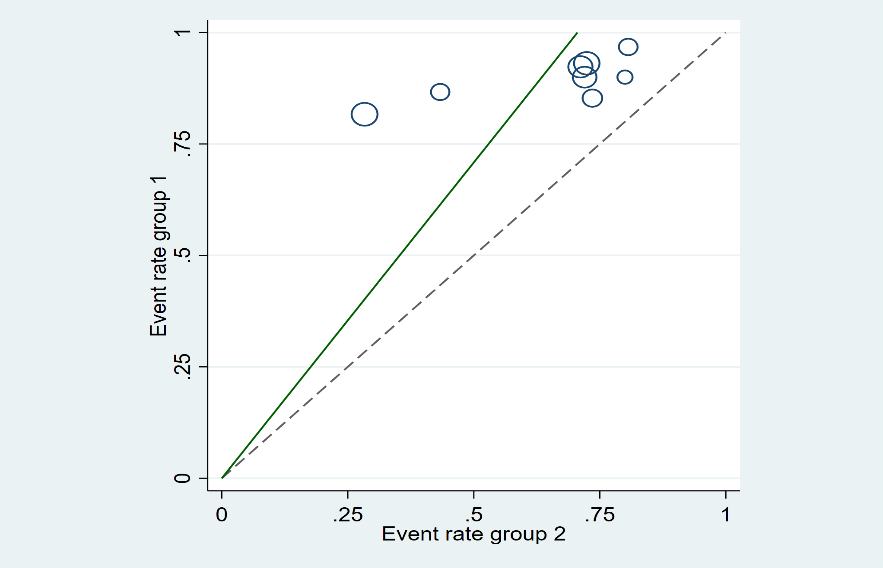


s Fig. 6 Galbraith radial plot comparing the effects of acupoint therapies plus other therapies versus other therapies alone on effective rates.


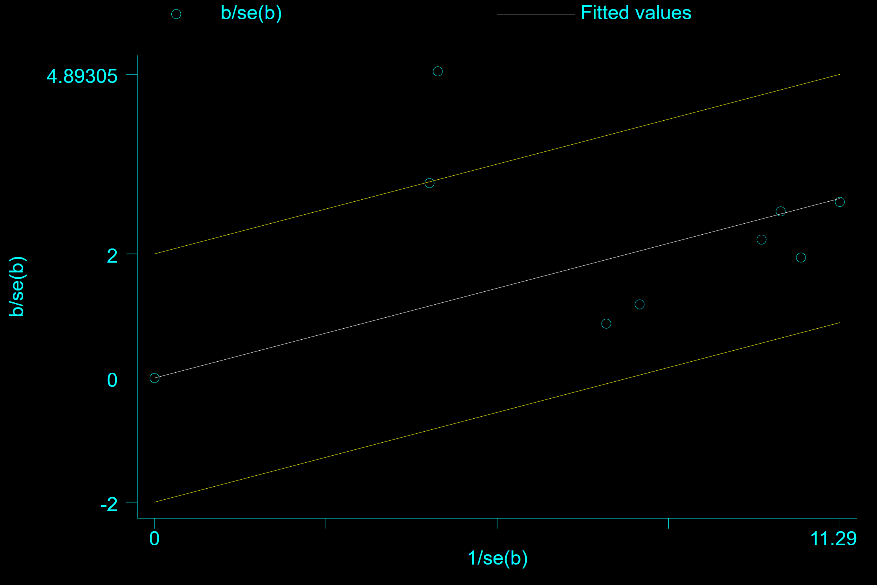


s Fig. 7 Forest plot comparing the effects of acupoint therapies plus other therapies versus other therapies alone on effective rates after removing one study.


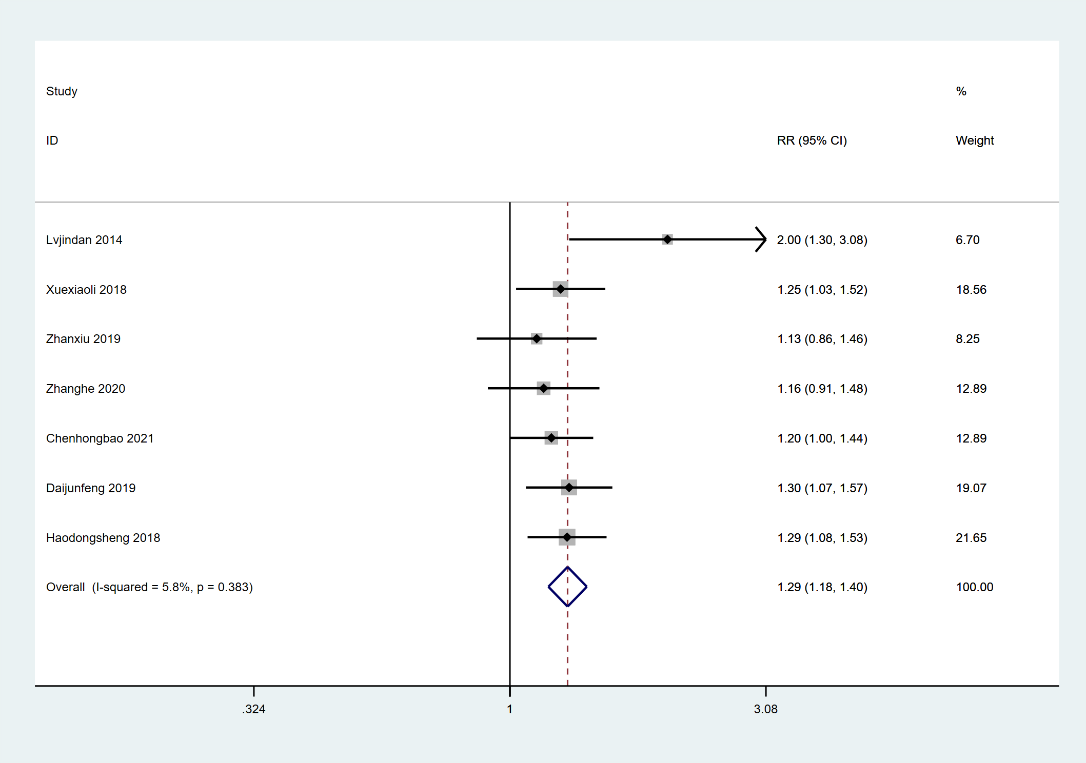


s Fig. 8 Funnel plot comparing the effects of acupoint therapies plus other therapies versus other therapies alone on effective rates after removing one study.


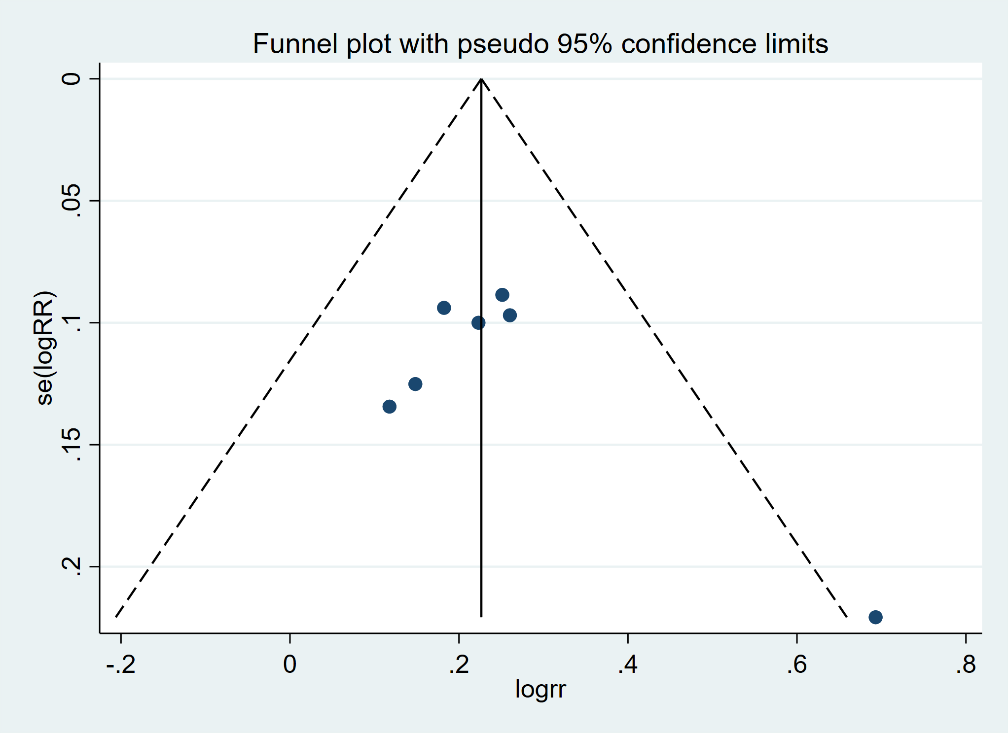


s Fig. 9 Egger’s bias test comparing the effects of acupoint therapies plus other therapies versus other therapies alone on effective rates after removing one study.

**
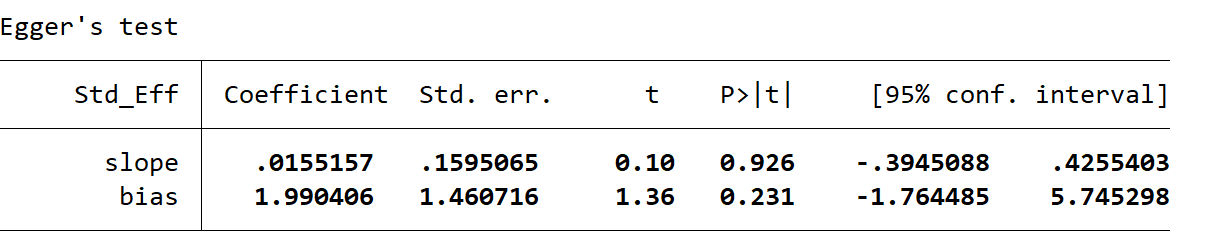
Note:** s Fig. 8 shows a decrease in heterogeneity after the removal of one paper with a greater impact on heterogeneity. The funnel plot and Egger’s bias test of eight studies indicated there was no publication bias (s Fig. 8 and s Fig. 9).

s Fig. 10 Forest plot comparing the effects of acupoint therapies plus CM or NDT versus CM or NDT alone on HAMD.


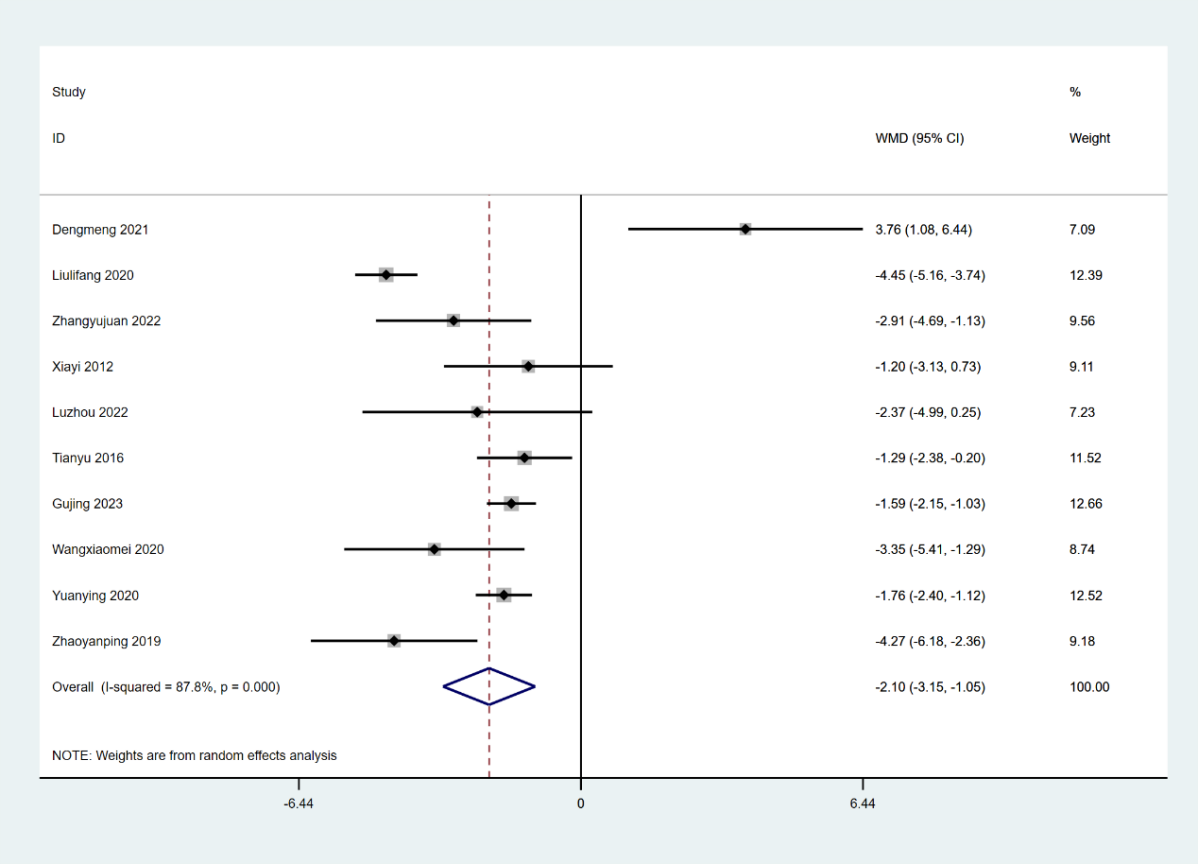


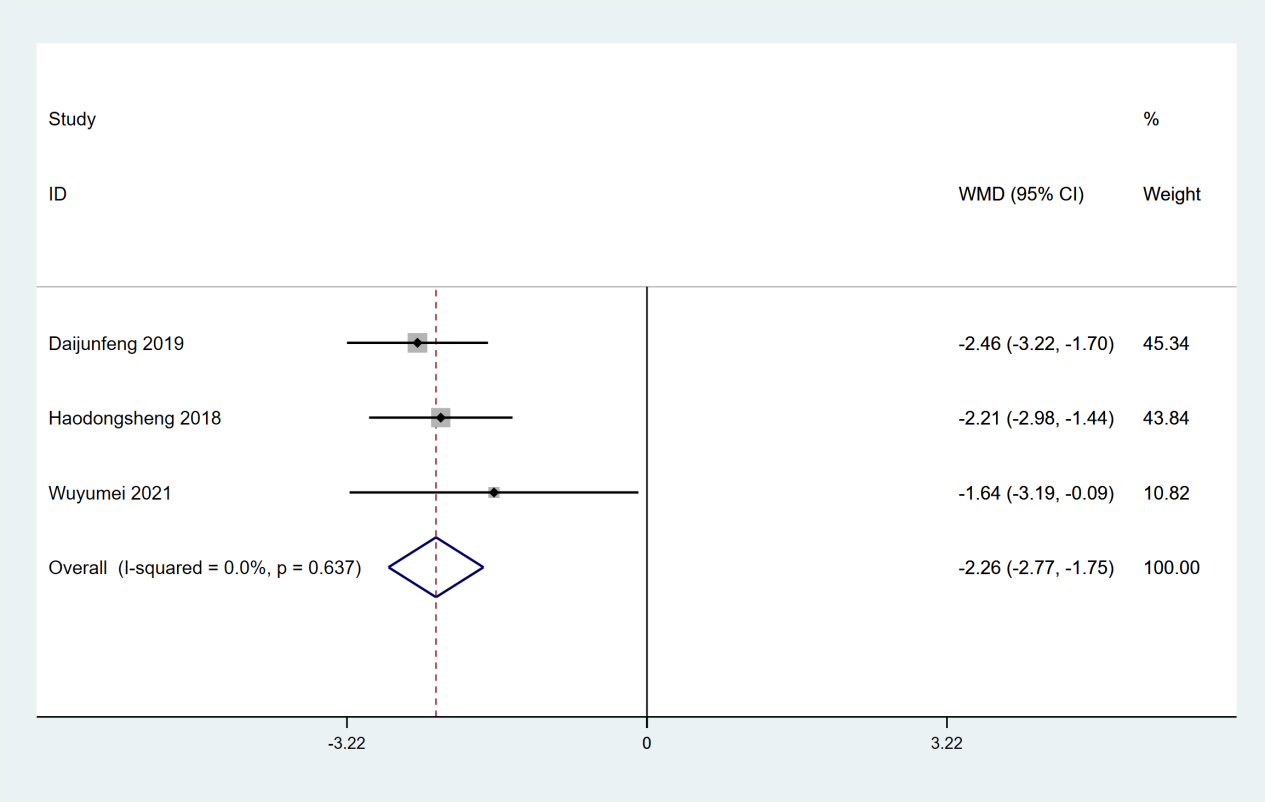
 s Fig. 11 Forest plot comparing the effects of acupoint therapies with other therapies versus other therapies alone on HAMD.

s Fig. 12 Meta-regression analysis of different acupoint therapies on HAMD.

s Fig.
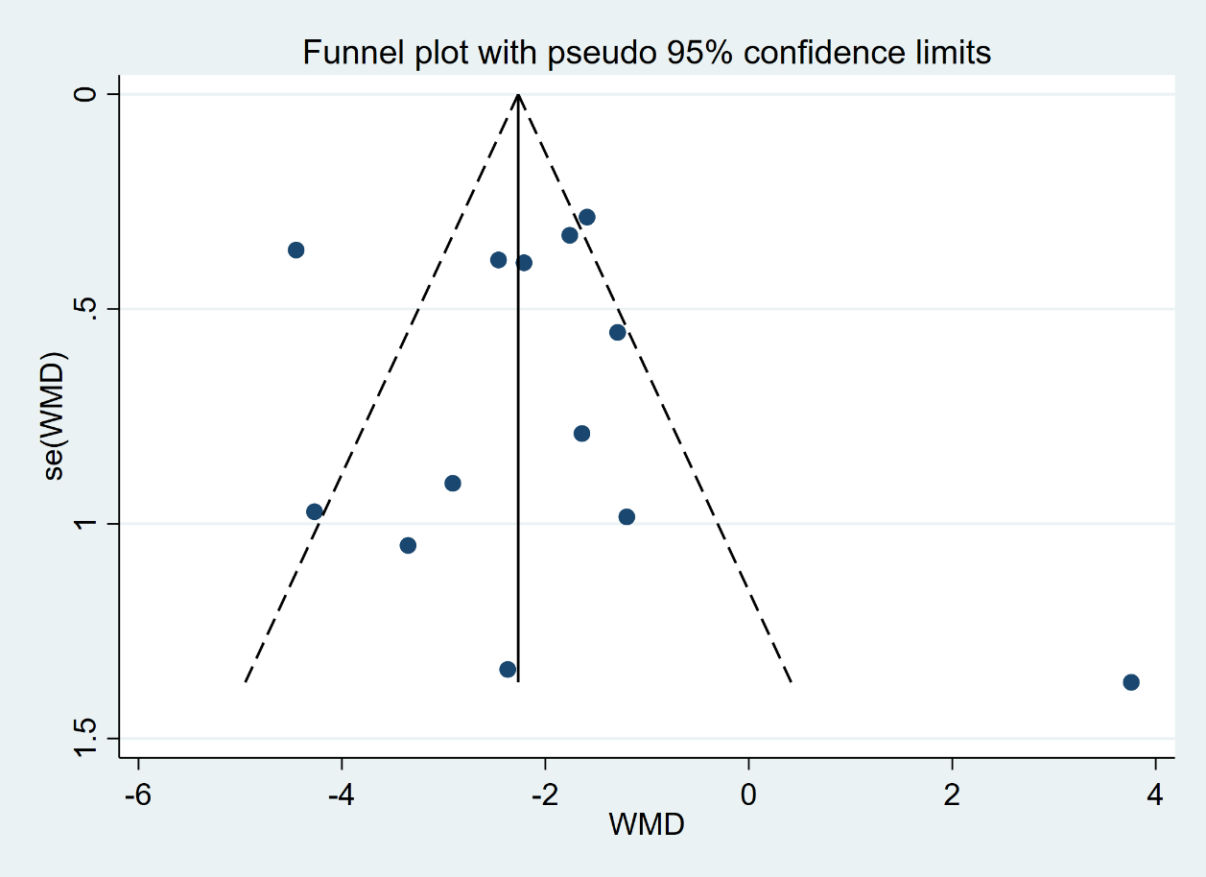

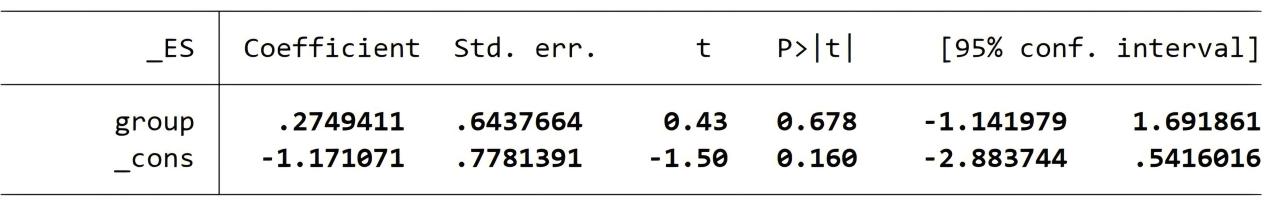
13 Funnel plot comparing the effects of acupoint therapies plus CM or NDT versus CM or NDT alone on HAMD.

s Fig. 14 Egger’s test comparing the effects of acupoint therapies plus CM or NDT versus CM or NDT alone on HAMD.


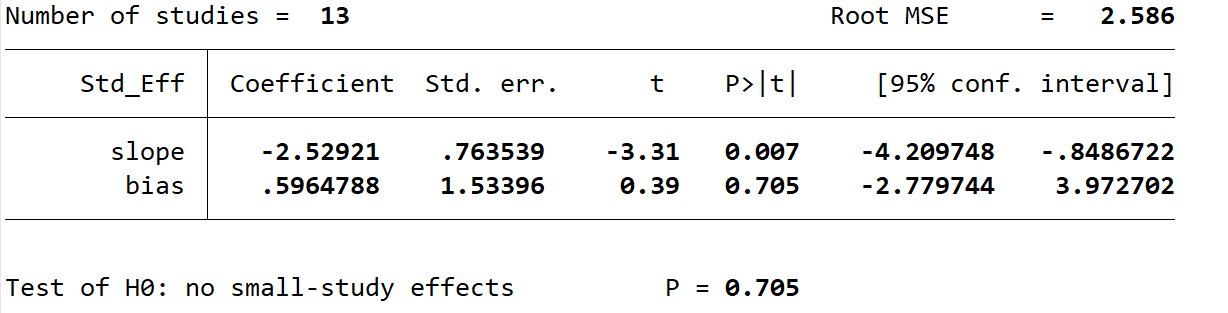


**Note:** Subgroup analysis was conducted. s Fig. 10 showed 10 studies reporting acupoint therapy with drugs versus drugs alone has strong heterogeneity after deleting 2 studies. s Fig. 11 showed there is no heterogeneity of the three studies reporting acupoint therapy combined with other therapies versus other therapies alone. s Fig. 12 showed meta-regression analysis effect of different acupoint therapies used “type of therapy” as an ordinal variable between acupuncture and moxibustion or acupoint sticking. s Fig. 13 and s Fig. 14 indicated there is no publication bias via funnel plot and Egger’s test.

s Fig. 15 Meta-regression analysis of different acupoint therapies on MoCA.


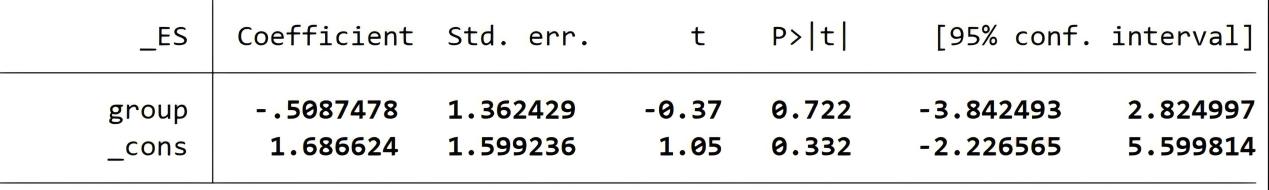


s Fig. 16 Meta-regression analysis of different acupoint therapies on PDSS.


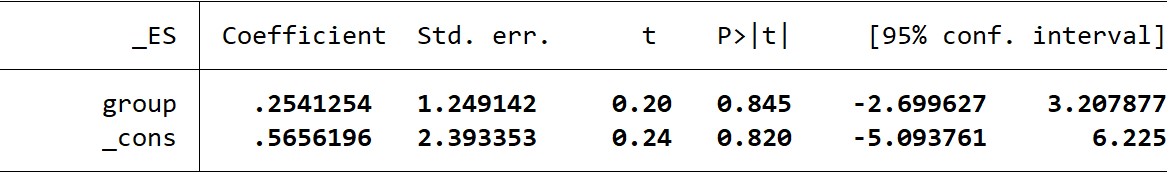


s Fig. 1
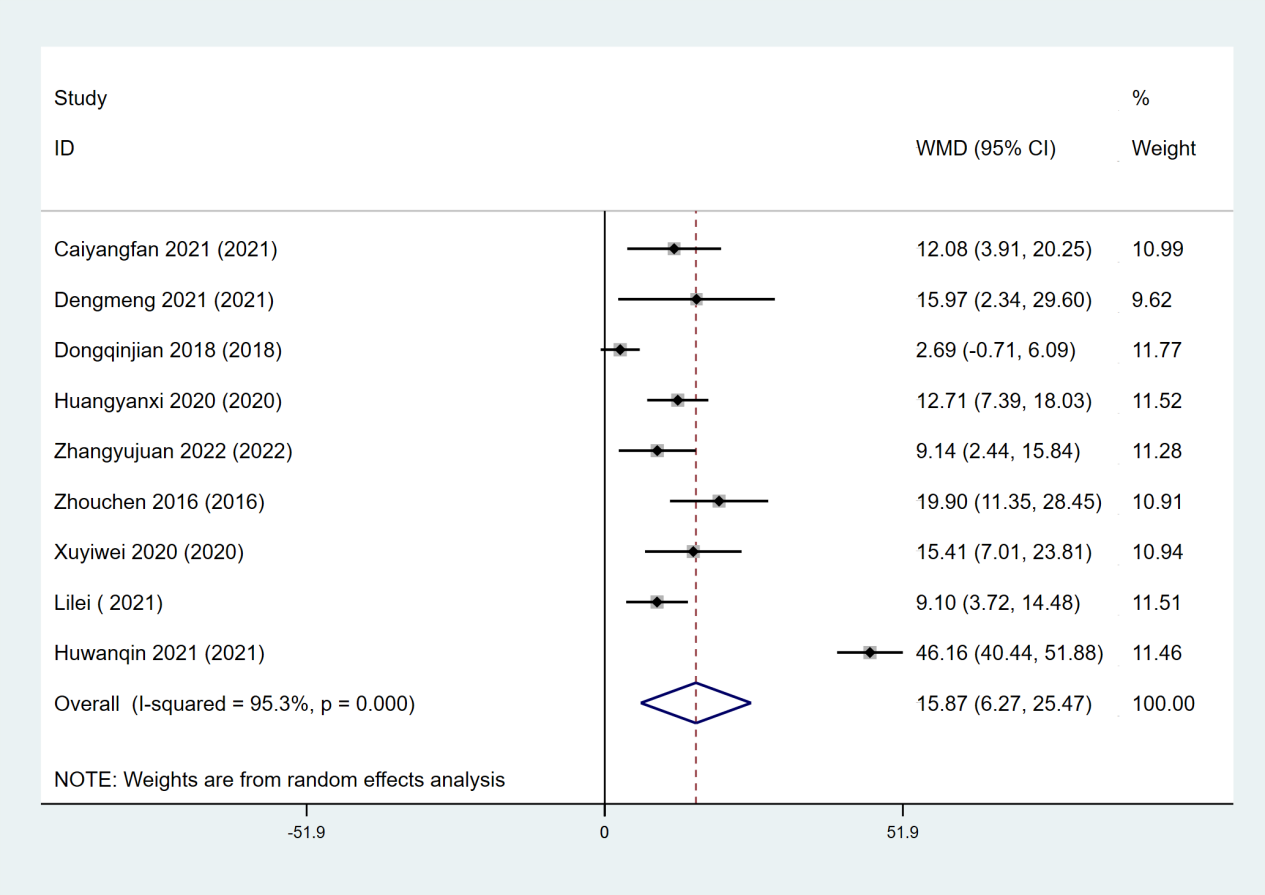
7 Funnel plot comparing the effects of acupoint therapies with drugs versus drugs alone on PDSS.


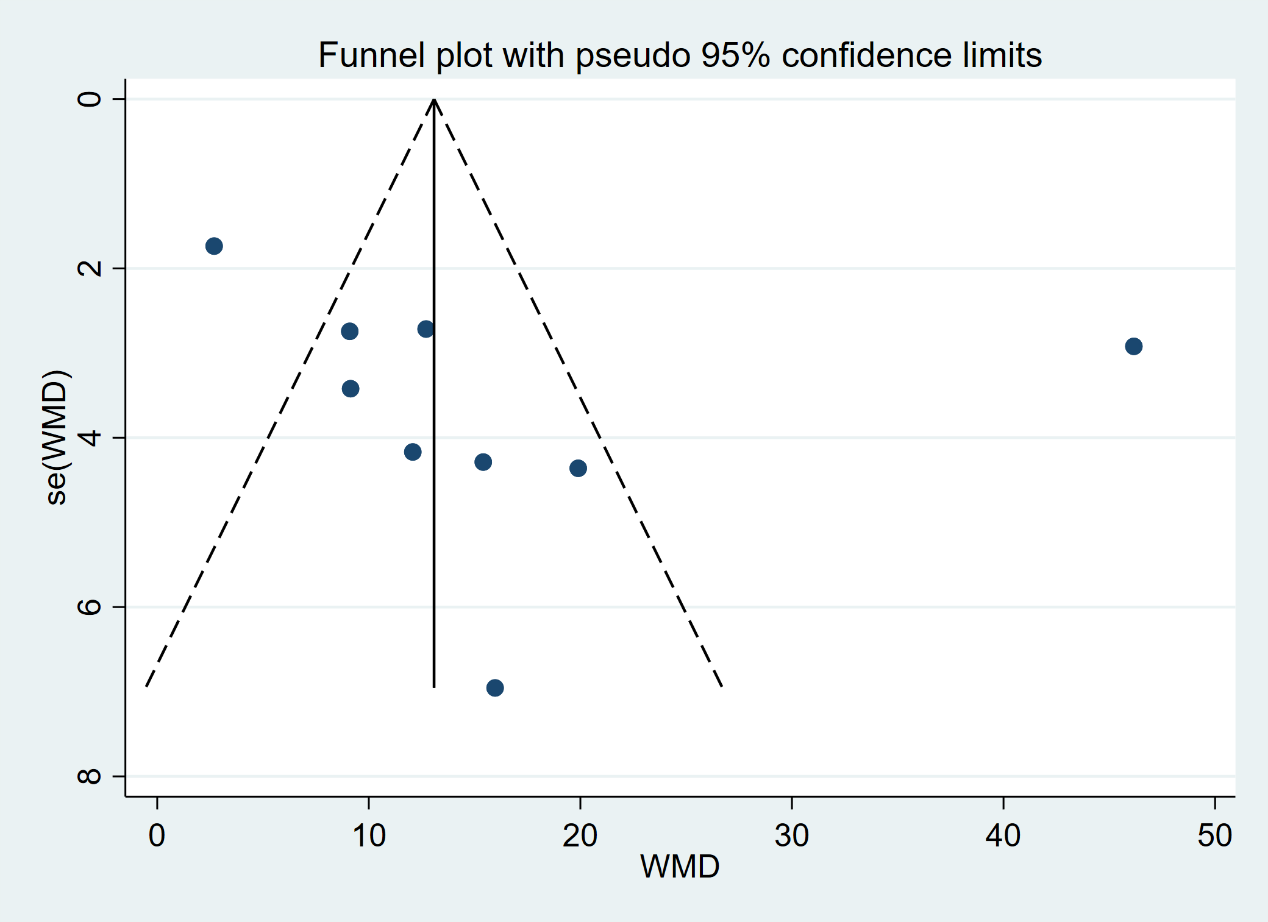


s Fig. 18 Egger’s bias test comparing the effects of acupoint therapies with drugs versus drugs alone on PDSS.


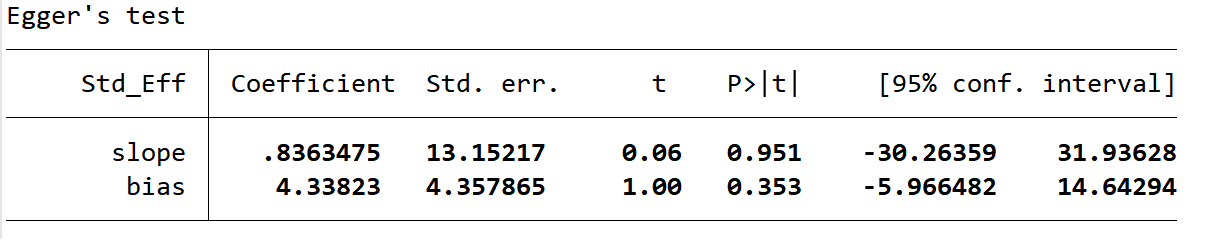


**Note:** Meta-regression analysis was conducted using used “type of therapy”, showing there were no differences between acupuncture and acupoint sticking (s Fig. 16) . s Fig. 17 and s Fig. 18 showed there were no publication bias via funnel plot and Egger’s bias test.

s Fig. 19 Meta-regression analysis of different acupoint therapies.


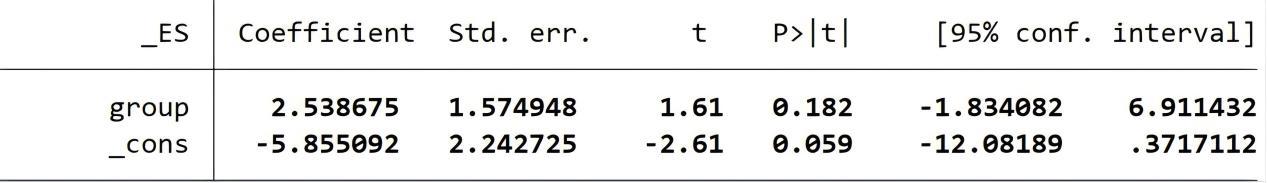


s Fig. 20 Funnel plot test comparing the effects of acupoint therapies with other therapies versus other therapies alone on PSQI.


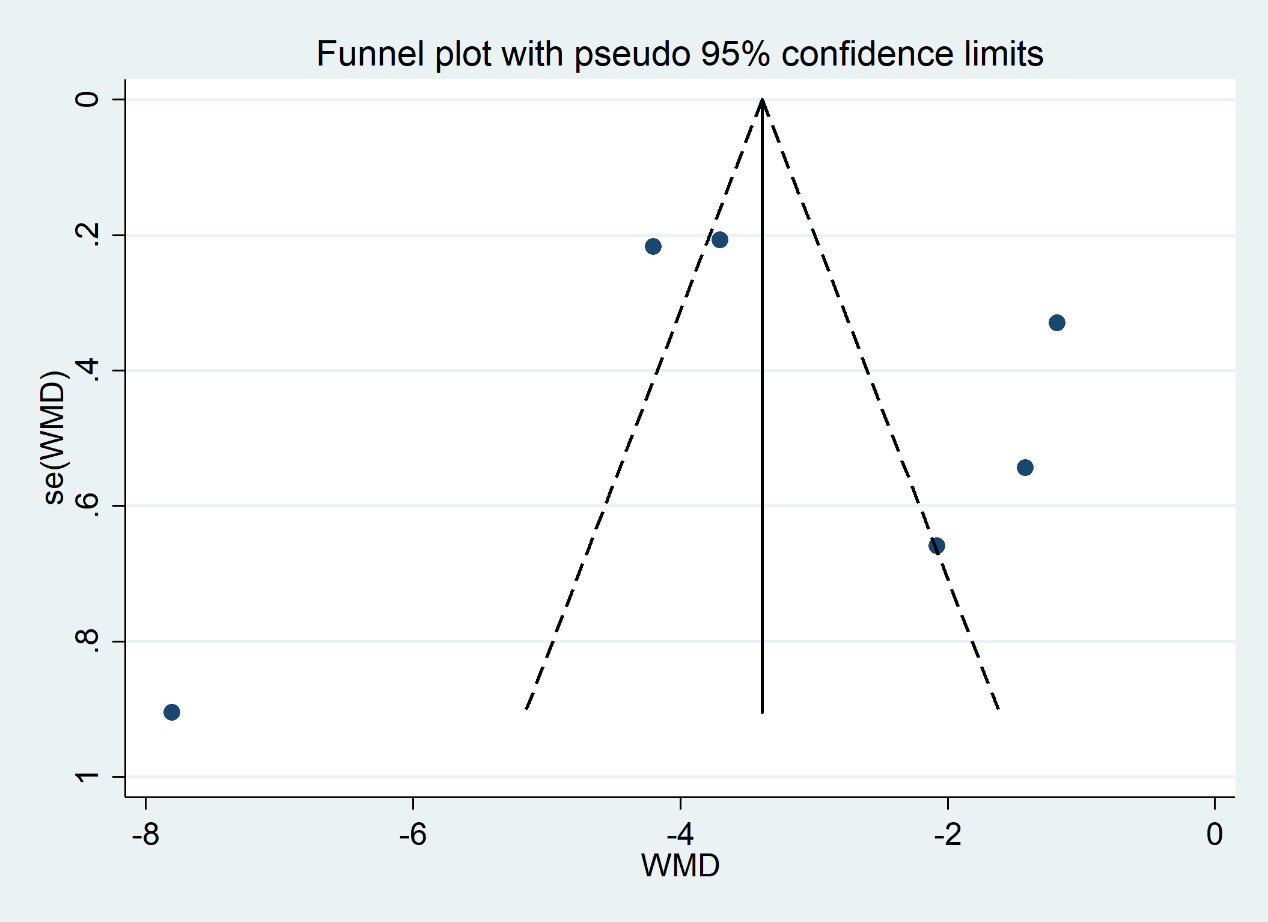


s Fig. 21 Egger’s bias test comparing the effects of acupoint therapies with other therapies versus other therapies alone on PSQI.


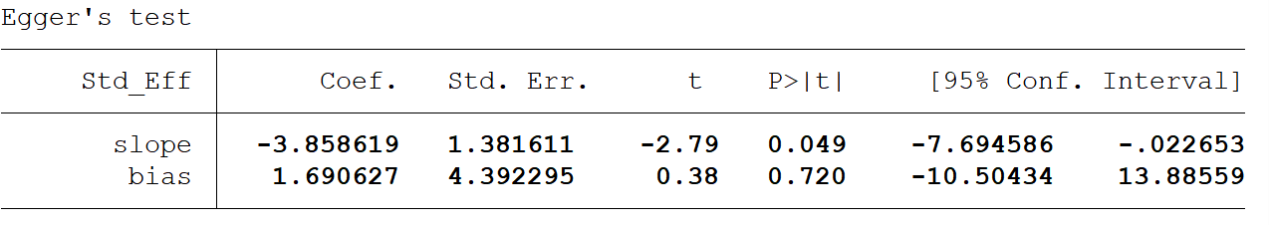


**Note:** Meta-regression analysis was conducted using used “type of therapy”, showing there were no differences between acupuncture and acupoint sticking (s Fig. 19) . s Fig. 20 and s Fig. 21 showed there were no publication bias via funnel plot and Egger’s bias test.

s Fig. 22 Meta-regression analysis of different acupoint therapies on PAC-QoL.


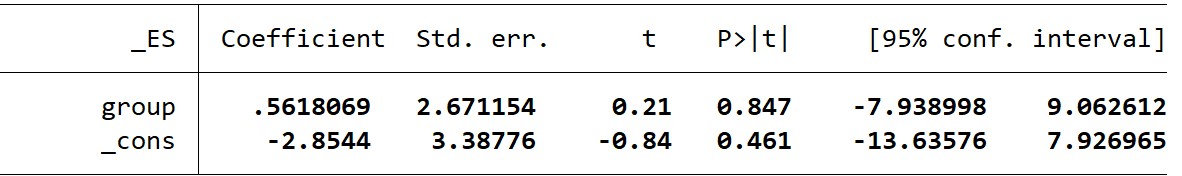


s Fig. 23 Egger’s bias test comparing the effects of acupoint therapies with other therapies versus other therapies alone on PAC-QoL scores.

**
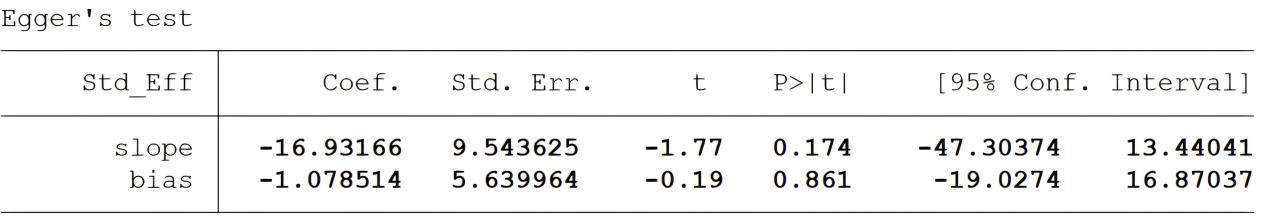
**

**Note：**Meta-regression analysis was conducted using used “type of therapy”, showing there were no differences between acupoint therapy with other therapies and other therapies alone (s Fig. 22) . s Fig. 23 showed there were no publication bias Egger’s bias test.
