# Supplementary material for: Combination treatment with acupoint therapy and conventional medication for non-motor symptoms in Parkinson’s disease: a systematic review and meta-analysis
Source: Front Neurol. 2025 May 22;16:1381500. doi: 10.3389/fneur.2025.1381500 (PMC12137075; doi:10.3389/fneur.2025.1381500)
Supplement: Supplementary file 3 [file Data_Sheet_3.docx]

**Search strategy**

1. **Search strategy for PubMed**

| **NO.** | **Search terms** |
| --- | --- |
| #1 | Acupoint. Mesh |
| #2 | Acupuncture Points. Mesh |
| #3 | （Acupuncture Point) or (Point, Acupuncture) or (Acupoints) or (Acupoint). ti. ab |
| #4 | Acupuncture. Mesh |
| #5 | Acupuncture. ti. ab |
| #6 | Acupressure. Mesh |
| #7 | Acupressure. ti. ab |
| #8 | Acupuncture, auricular. Mesh |
| #9 | (Acupunctures, Ear) or (Ear Acupunctures) or (Auricular Acupuncture) or (Acupuncture, Auricular) or (Auricular Acupunctures). ti. ab |
| #10 | Massage. Mesh |
| #11 | Massage. ti. ab |
| #12 | (Manipulations, Musculoskeletal) or (Manipulation Therapy) or (Manipulative Therapies) or (Therapies, Manipulative) or (Therapy, Manipulation) or (Manipulation Therapies) or (Manual Therapies) or (Therapy, Manual). ti. ab |
| #13 | Cupping therapy. Mesh |
| #14 | (Cupping Therapies) or (Therapy, Cupping) or (Cupping Treatment) or (Treatment, Cupping). ti. ab |
| #15 | Moxibustion. Mesh |
| #16 | Moxibustion. ti. ab |
| #17 | Acupoint injection. Mesh |
| #18 | Acupoint injection.ti. ab |
| #19 | #1 or #2-18 |
| #20 | Randomized controlled trial. Mesh |
| #21 | Controlled clinical trial. ti. ab |
| #22 | Randomized. ti. ab |
| #23 | Randomly. ti. ab |
| #24 | Trial. ti. ab |
| #25 | #20 or #21-24 |
| #26 | Parkinson’s disease. Mesh |
| #27 | Parkinson’s disease. ti. ab |
| #28 | Parkinsonian disorders. ti. ab |
| #29 | #26 or #27-28 |
| #30 | #19 and #25 and #29 |

1. **Search strategy for Web of Science**

#1 TS=(Parkinson's Disease OR Idiopathic Parkinson's Disease OR Lewy Body Parkinson's Disease OR Parkinson's Disease, Idiopathic OR Parkinson's Disease, Lewy Body OR Parkinson Disease, Idiopathic OR Parkinson's Disease OR Idiopathic Parkinson Disease OR Lewy Body Parkinson Disease OR Primary Parkinsonism OR Parkinsonism, Primary OR Paralysis Agitans)

#2 TS=(acupuncture OR moxibustion OR moxabustion OR Acupuncture Point OR Point, Acupuncture OR Points, Acupuncture OR Acupoints OR Acupoint OR Acupunctures, Ear OR Ear Acupunctures OR Auricular Acupuncture OR Ear Acupuncture OR Acupuncture, Auricular OR Acupunctures, Auricular OR Auricular Acupunctures OR Zone Therapy OR Therapies, Zone OR Zone Therapies OR Therapy, Zone OR Massage Therapy OR Massage Therapies OR Therapies, Massage OR Therapy, Massage OR Shiatsu OR Zhi Ya OR Chih Ya OR Shiatzu OR Acupoint injection)

#3 TS= (Randomized controlled trial OR Controlled trial OR randomized)

#4 #1 AND #2 AND #3

1. **Search strategy for Cochrane Library**

#1  MeSH descriptor: [Parkinson Disease] explode all trees

#2 (Idiopathic Parkinson's Disease):ti,ab,kw OR (Lewy Body Parkinson's Disease):ti,ab,kw OR (Parkinson's Disease, Idiopathic)ti,ab,kw OR (Parkinson's Disease, Lewy Body):ti,ab,kw OR (Parkinson Disease, Idiopathic):ti,ab,kw OR (Parkinson's Disease):ti,ab,kw OR (Idiopathic Parkinson Disease):ti,ab,kw OR (Lewy Body Parkinson Disease):ti,ab,kw OR (Primary Parkinsonism):ti,ab,kw OR (Parkinsonism, Primary):ti,ab,kw OR (Paralysis Agitans):ti,ab,kw

#3 #1 OR #2

#4 MeSH descriptor: [Acupuncture] explode all trees

#5 MeSH descriptor: [Acupressure] explode all trees

#6 MeSH descriptor: [Acupressure, Ear] explode all trees

#7 MeSH descriptor: [Massage] explode all trees

#8 MeSH descriptor: [Cupping Therapy] explode all trees

#9 MeSH descriptor: [Moxibustion] explode all trees

#10 MeSH descriptor: [Acupoint injection)] explode all trees

#11 (acupuncture):ti,ab,kw OR (moxabustion):ti,ab,kw OR (moxibustion):ti,ab,kw OR(Acupuncture point):ti,ab,kw OR (Point,Acupuncture):ti,ab,kw OR(Points,Acupuncture):ti,ab,kw OR (Acupoints):ti,ab,kw OR (Acupoint):ti,ab,kw OR(AcupuncturesEar):ti,ab,kw OR(Ear Acupunctures):ti,ab,kw OR (Auricular Acupuncture):ti,ab,kw OR (Ear Acupuncture):ti,ab,kw OR (Acupuncture, Auricular):ti,ab,kw OR(Acupunctures,Auricular):ti,ab,kw OR(Auricular Acupunctures):ti,ab,kw OR (Zone Therapy):ti,ab,kw OR (Therapies,Zone):ti,ab,kw OR(Zone Therapies):ti,ab,kw OR (Therapy, Zone):ti,ab,kw OR (Massage Therapy):ti,ab,kw OR (Massage Therapies):ti,ab,kw OR (Therapies, Massage):ti,ab,kw OR(Therapy, Massage):ti,ab,kw OR (Shiatsu):ti,ab,kw OR (Zhi Ya):ti,ab,kw OR(Chih Ya):ti,ab,kw OR (Shiatzu):ti,ab,kw OR (Acupoint injection):ti,ab,kw

#12 #4 OR #5 OR #6 OR #7 OR #8 OR #9 OR #10 OR #11

#13 MeSH descriptor: [Randomized Controlled Trials as Topic] explode all trees

#14 ("Randomized Controlled Trial"):ti,ab,kw OR ("Randomized"):ti,ab,kw OR

("Controlled"):ti,ab,kw

#15 #13 AND #14

#16 #3 AND #12 AND #15

1. **Search strategy for Embase**

#1 Parkinson disease /exp

#2 'parkinson disease, lewy body':ab,ti OR 'parkinson disease, idiopathic':ab,ti OR 'parkinson disease':ab,ti OR 'idiopathic parkinson disease':ab,ti OR 'lewy body parkinson disease':ab,ti OR 'parkinsonism, primary':ab,ti OR 'primary parkinsonism':ab,ti OR 'paralysis agitans':ab,ti

#3 #1 OR #2

#4 'acupressure'/exp

#5 'acupuncture point'/exp

#6 'acupuncture'/exp

#7 'auricular acupuncture'/exp OR 'auricular acupuncture'

#8 'massage'/exp

#9 'cupping therapy'/exp

#10 'moxibustion'/exp

#11 'acupuncture':ab,ti OR 'moxibustion':ab,ti OR 'moxabustion':ab,ti OR 'acupuncture point':ab,ti OR 'point, acupuncture':ab,ti OR 'points, acupuncture':ab,ti OR 'acupoints':ab,ti OR 'acupoint':ab,ti OR 'acupunctures, ear':ab,ti OR 'ear acupunctures':ab,ti OR 'auricular acupuncture':ab,ti OR 'ear acupuncture':ab,ti OR 'acupuncture, auricular':ab,ti OR 'acupunctures, auricular':ab,ti OR 'auricular acupunctures':ab,ti OR 'zone therapy':ab,ti OR 'therapies, zone':ab,ti OR 'zone therapies':ab,ti OR 'therapy, zone':ab,ti OR 'massage therapy':ab,ti OR 'massage therapies':ab,ti OR 'therapies, massage':ab,ti OR 'therapy, massage':ab,ti OR 'shiatsu':ab,ti OR 'zhi ya':ab,ti OR 'chih ya':ab,ti OR 'shiatzu':ab,ti

#12 #4 OR #5 OR #6 OR #7 OR #8 OR #9 OR #10 OR #11

#13 'randomized controlled trial'/exp OR 'controlled clinical trial'/exp

#14 'randomized controlled trial':ab,ti OR 'controlled clinical trial':ab,ti

#15 #13 OR #14

#16 #3 AND #12 AND #15

1. **Search strategy for CNKI**

(SU=帕金森病 OR SU=老年人帕金森病 OR SU=老年人帕金森病 OR SU=帕金森氏症) AND (SU=针灸OR SU=针法 OR SU=针刺疗法 OR SU=电针 OR SU=耳针 OR SU=刺法 OR SU=灸法 OR SU=穴位按压 OR SU=推拿 OR SU=拔罐 OR SU=穴位注射) AND (SU=随机 OR SU=随机对照 OR SU= RCT OR FT=随机)

1. **Search strategy for Wanfang Database**

主题： (帕金森病 or 帕金森氏症 or 帕金森 or 颤证 or 震颤麻痹) and 主题：(针灸 or 电针 or 耳针 or 穴位 or 针刺 or 灸法 or 穴位按压 or 推拿 or 穴位注射 or 拔罐) and 主题：(随机对照)

1. **Search strategy for VIP**

M=(帕金森病 or 帕金森氏症 or 帕金森 or 颤证 or 震颤麻痹) AND M=(针灸 or 电针 or 耳针 or 穴位 or 针刺 or 灸法 or 推拿 or 拔罐 or 穴位按压 or 穴位注射) AND M=(RCT or 随机对照 or 随机分配 or 随机)

1. **Search strategy for CBM**

#1主题词=帕金森病/全部副主题词

#2 帕金森病 OR 帕金森氏症 OR 帕金森 OR 颤证 OR 震颤麻痹

#3 #1 OR #2

#4 主题词= 针灸疗法 OR 针灸疗法 OR 穴位疗法

#5 针刺 OR 刺法 OR灸法 OR 穴位注射 OR 电针 OR 针刺 OR 耳针 OR 推拿 穴位按压

#6 #4 OR #5

#7 主题词=随机对照试验[文献类型]

#8主题词=随机分配

#9主题词=随机对照试验

#10 #7 OR #8 OR #9

#11 3# AND #6 AND #10
